# Progression of Osteosarcoma from a Non-Metastatic to a Metastatic Phenotype Is Causally Associated with Activation of an Autocrine and Paracrine uPA Axis

**DOI:** 10.1371/journal.pone.0133592

**Published:** 2015-08-28

**Authors:** Liliana Endo-Munoz, Na Cai, Andrew Cumming, Rebecca Macklin, Lilia Merida de Long, Eleni Topkas, Pamela Mukhopadhyay, Michelle Hill, Nicholas A Saunders

**Affiliations:** The University of Queensland Diamantina Institute, Translational Research Institute, Brisbane, Queensland 4102, Australia; Johns Hopkins University, UNITED STATES

## Abstract

Pulmonary metastasis is the major untreatable complication of osteosarcoma (OS) resulting in 10–20% long-term survival. The factors and pathways regulating these processes remain unclear, yet their identification is crucial in order to find new therapeutic targets. In this study we used a multi-omics approach to identify molecules in metastatic and non-metastatic OS cells that may contribute to OS metastasis, followed by validation *in vitro* and *in vivo*. We found elevated levels of the urokinase plasminogen activator (uPA) and of the uPA receptor (uPAR) exclusively in metastatic OS cells. uPA was secreted in soluble form and as part of the protein cargo of OS-secreted extracellular vesicles, including exosomes. In addition, in the tumour microenvironment, uPA was expressed and secreted by bone marrow cells (BMC), and OS- and BMC-derived uPA significantly and specifically stimulated migration of metastatic OS cells *via* uPA-dependent signaling pathways. Silencing of *uPAR* in metastatic OS cells abrogated the migratory response to uPA *in vitro* and decreased metastasis *in vivo*. Finally, a novel small-molecule inhibitor of uPA significantly (*P* = 0.0004) inhibited metastasis in an orthotopic mouse model of OS. Thus, we show for the first time that malignant conversion of OS cells to a metastatic phenotype is defined by activation of the uPA/uPAR axis in both an autocrine and paracrine fashion. Furthermore, metastasis is driven by changes in OS cells as well as in the microenvironment. Finally, our data show that pharmacological inhibition of the uPA/uPAR axis with a novel small-molecule inhibitor can prevent the emergence of metastatic foci.

## Introduction

Osteosarcoma (OS) is the most commonly diagnosed paediatric primary bone malignancy [1]. The most frequent complication is the development of metastatic disease [2], with up to 80% of patients having clinically undetectable metastasis at the time of diagnosis [3]. Treatment involving intensive multi-agent neo-adjuvant chemotherapy has increased the 5-year survival of patients with localized tumours to 65–75% [3–6]. In contrast, patients with metastatic disease remain refractory to chemotherapy and have a 5-year survival of only 10–20% [2, 7]. Thus, novel therapies, which may be used either alone or in combination with current systemic treatment, are needed in order to improve the survival of patients with metastatic OS.

We have previously shown that the metastatic behavior of OS is dictated by a combination of tumour cell-specific (inherent) and microenvironmental factors [8]. For example, comparison of a suite of established human OS cell lines in an orthotopic mouse model reveals that OS cell lines can be categorized as inherently metastatic or non-metastatic. In the clinical setting, there is a cohort of patients that will not develop metastatic disease, and transcriptomic analyses of chemo-naïve patient biopsies have identified gene and mRNA/miRNA signatures that predict the development of chemo-resistant metastatic disease.at the time of diagnosis [8, 9]. In addition, microenvironmental factors such as loss of osteoclasts and OS cell sensitivity to bone marrow cell (BMC)-secreted factors regulate OS cell migration and metastasis [8]. However, the specific factors regulating inherent and BMC-stimulated metastasis have not been identified.

The aim of this study was to identify factors which may regulate OS cell migration and metastasis, and which could then become potential therapeutic targets. To this end, we performed a comparative multi-omics analysis using a representative cohort of human OS cell lines which are consistently metastatic or non-metastatic *in vivo*. The results of the combined transcriptomic, proteomic and secretomic analysis identified the urokinase plasminogen activator (uPA) and its receptor (uPAR) as key molecules associated with OS metastasis.

The urokinase plasminogen activator is a serine protease involved in extracellular matrix (ECM) degradation, and plays a significant role in the progression and metastasis of a number of solid tumours including breast, lung, prostate, pancreas, ovary, kidney, and colon [10]. Binding of active uPA to uPAR initiates a proteolytic cascade that results in the conversion of plasminogen to plasmin, ECM degradation and activation of matrix metalloproteinases which facilitate cell invasion and metastasis [10]. In addition, uPA-uPAR binding can invoke non-proteolytic receptor-dependent signalling pathways that modulate migration, adhesion, differentiation, proliferation and survival, that contribute to tumour progression and angiogenesis [11–13]. Thus, the effects of the uPA-uPAR axis can be mediated *via* activation of proteolysis or signalling pathways or both. However, until recently, it has been difficult to discriminate between the actions of the two pathways. Moreover, pharmacological inhibitors of the uPA/uPAR axis have not been available for clinical use.

The results of our multi-omics analysis are supported by early studies reporting that uPA expression correlated with an invasive or metastatic phenotype in human OS [14–19]. However, although these studies provided initial evidence for the potential importance of the uPA/uPAR axis in OS growth and metastasis, they were limited to the use of a rat OS cell line and did not address the mechanism by which uPA/uPAR contributed to metastasis. In this study, we make several novel findings: (i) We show that activation of the uPA/uPAR axis is diagnostic of the conversion of OS cells from a non-metastatic to a metastatic phenotype. (ii) We provide evidence to show that the uPA/uPAR axis is activated in metastatic OS cells *via* an autocrine loop, (iii) that the uPA/uPAR axis is further stimulated, in a paracrine fashion, by stromal cells, (iv) that the driver of uPA-mediated metastasis is the dysregulated and selective overexpression of uPAR in metastatic OS cells, v) that uPA-dependent OS metastasis can be mediated *via* uPA-dependent signalling events such as MAPK, independently of the proteolytic activity of uPA, (vi) that human OS cells secrete both an active “free” form of uPA as well as an extracellular vesicle (exosome) encapsulated form of uPA and finally, (vii) we show that targeted inhibition of the uPA/uPAR axis with a new and selective pharmacological inhibitor significantly reduces OS pulmonary metastasis in a preclinical model of OS.

## Materials and Methods

### Tissue culture, cell lines, subclones and bone marrow cells

Osteosarcoma cells, KHOS (ATCC CRL-1544), KRIB [20], HOS (ATCC CRL-1543), U2OS (ATCC HTB-96), BTK143B (ATCC CRL-8303), SJSA (ATCC CRL-2098), SaOS (ATCC HTB-85), G292 (ATCC CRL-1423), and MG63 (ATCC CRL-1427), were a kind gift from Prof. Andreas Evdokiou, Basil Hetzel Institute, University of Adelaide, Australia, and Dr Steve Bouralexis, Prince Henry’s Institute, Melbourne, Australia. Cells were cultured in DMEM/10% FCS. All cell lines were authenticated by Cell Bank Australia using PCR to identify short tandem repeats and match them to known ATCC profiles. The highly metastatic subclone, C6, and the poorly metastatic subclone, C5, were isolated from KHOS. Cells were seeded in 24-well plates at a density of less than 1 cell/well. Individual cells were allowed to divide and grow into colonies over several days. Wells that contained expanding colonies were expanded into tissue culture flasks and frozen in liquid nitrogen. Subclones were selected at random and injected intrafemorally into four female 6-week-old BALB/c nude mice as described elsewhere in this section. KHOS cells were injected as a control. Collection of tissue and quantification of lung metastases is described elsewhere in this section. Bone marrow cells (BMC) were collected by flushing cavities of mouse femurs with MEM/10% FCS. BMC were centrifuged, resuspended in MEM/10% FCS, and plated at 5x10^5^ cells/cm^2^ in MEM/10% FCS supplemented with 20 ng/mL CSF-1 (Peprotech, NJ, USA). Medium was changed on day 3 and collected on day 6. The latter was clarified by centrifugation, filtered, and stored at -20°C.

### Transcriptomic analysis

Metastatic (KHOS, KRIB) and non-metastatic (HOS, U2OS) OS cells were analyzed individually in triplicate on a HumanHT-12 v4 Expression BeadChip (Illumina, Melbourne, Australia) after amplification using the Illumina TotalPrep RNA Amplification Kit (Life Technologies, Melbourne, Australia). Raw gene expression values for each cell line were extracted with BeadStudio data analysis software (Illumina, Melbourne, Australia). Data quality was determined using the positive and negative control probes as well as by inspection of the distributions of probe intensities. Data were normalized using the quantile normalization method in the Lumi package of Bioconductor. Probes that were called present (detection P < 0.1) in at least 5 samples were retained. A moderated Student t test in the Limma package of Bioconductor was applied to test differential expression, and FDR adjustment of the P value was performed to correct for multiple testing. To find differentially-expressed genes between metastatic and non-metastatic cells, the normalized expression values for the metastatic cells (KHOS+KRIB) were combined and compared to the combined normalized expression values of the non-metastatic cells (HOS+U2OS). Probes were considered significantly different if adjusted P value was < 0.1, and fold-change difference between groups was at least 1.5. (NCBI GEO accession No.GSE49003). Stringent analysis using a B-statistic > 3 was used to select genes that had ≥ 95% chance of being differentially expressed between the metastatic and non-metastatic groups. Gene enrichment analysis was performed using Ingenuity Pathway Analysis (Ingenuity Systems, CA, USA).

### Proteomic analysis

We used stable isotope labelling with amino acids in cell culture (SILAC) [21]. Plasma membrane proteins, or proteins secreted into serum-reduced medium of metastatic (KHOS, KRIB) and non-metastatic (HOS, U2OS) OS cells were labelled with “heavy” (^13^C_6_^15^N_2_-Lys) and “light” (^13^C_6_^15^N_4_-Arg) amino acids (Silantes, Munich, Germany) respectively. Membrane and cytoplasmic fractions were separated by SDS-PAGE and bands excised for in-gel digestion. Tryptic peptides were subjected to LV-MS/MS using an Agilent 6520 QTOF coupled with a Chip CUBE and 1200 HPLC (Agilent Technologies, Melbourne, Australia). Raw data was processed as described elsewhere [22].

### Secretomic analysis

Medium was collected from 24 h cultures of KHOS, KRIB, HOS and U2OS cells grown in serum-reduced conditions. Medium was collected, centrifuged, filtered and incubated on a cytokine antibody array (RayBio Biotin Label-based Human Antibody Array 1, 507 proteins) (RayBiotech, GA, USA). ImaGene software (BioDiscovery Inc, CA, USA) was used to quantify the spot intensities on array membranes. Sample intensities were corrected against positive and negative controls on the array and expressed in arbitrary units.

### Exosome isolation and size measurement

Semi-confluent cultures of KHOS were grown for 24 hours in exosome-depleted DMEM/10% FCS. Medium was collected and sequentially centrifuged at 300 x *g/*10 min, 2000 x *g*/10 min, 10,000 x *g*/10 min, 100,000 x *g*/70 min, at 4°C. Exosome pellets were resuspended in cold PBS or RIPA buffer. Protein was determined by the method of Bradford [23] using a dye reagent concentrate (Bio-Rad, Sydney, Australia). Exosomes were stored at -80°C in 20 μg aliquots. Particle size analysis was performed in a Zetasizer Nano S (Malvern Instruments, UK).

### Western blot

KHOS, KRIB and BTK143B cells were seeded at equal concentrations and incubated with 100 nM uPA (Molecular Innovations, MI, USA) for 24 h. KHOS WT and KHOS uPAR-KD cells were seeded at equal concentrations and incubated with 100 nM rh-uPA (Molecular Innovations) for 24 h. Cells were washed with cold PBS and lysed in RIPA buffer with protein inhibitor cocktail (Thermo Fisher Scientific, IL, USA). Serum-reduced conditioned medium was concentrated in Amicon Ultra-15 Centrifugal Filter Units (Merck Millipore, Melbourne, Australia). Exosome pellets were resuspended in RIPA buffer as above. Protein assays were performed as above (BioRad). Samples were run on a 10% SDS-PAGE gel. Proteins were detected with monoclonal mouse anti-human uPAR (clone 109801) (R&D Systems), 1:500, under non-reducing conditions, mouse anti-human uPA (H-140), 1:200 (Santa Cruz Biotechnology, TX, USA), rabbit antibody to phospho-p44/42 MAPK (Erk1/2) (Thr202/Tyr204), 1:5000 (Cell Signaling Technology, MA, USA), rabbit antibody to Erk2 (C-14), 1:2000 (Santa Cruz), rabbit antibody to CD63, 1:1000 (EXOAB-CD63A-1, System Biosciences, CA, USA).

### Immunohistochemistry

Formalin-fixed, paraffin-embedded sections of mouse femurs, OS tumours and lungs were incubated with rabbit anti-human uPA (H-140) or goat anti-human uPAR (Santa Cruz), 1:100 and 1:200, respectively. Negative rabbit IgG (Dako, Melbourne, Australia) or negative goat IgG (Santa Cruz), were used as a controls. Quantitative image analysis was performed as described [24].

### Migration assays

These were performed as described [25], using uncoated membranes. OS cells (2 x 10^5^ cells/mL) were seeded in DMEM/10% FCS or in conditioned medium, with or without rh-uPA or recombinant murine uPA (rm-uPA) (Molecular Innovations), or neutralizing antibody to uPAR (American Diagnostica/Sekisui Diagnostics, MA, USA) or amiloride (Sigma-Aldrich, Sydney, Australia), into the upper chamber of Transwell plates (Corning Life Sciences, Melbourne, Australia). Cells were allowed to migrate for 24 h towards DMEM with 20% FCS in the lower chamber. Migrated cells were detected with 8 μM calcein-AM (Sigma-Aldrich) and detached with trypsin-EDTA. Fluorescence was measured in a FLUOstar Optima (BMG Labtech, Melbourne, Australia) at 485 nm (excitation) and 520 nm (emission).

### Cell proliferation assays

These were performed for 24 h at a cell concentration of 5 x 10^4^ cells/mL, using the CellTiter^96^ AQ_ueous_ One Solution Cell Proliferation Assay (Promega, Sydney, Australia).

### uPA activity assay

Cells were grown in phenol red-free DMEM/10% FCS. uPA activity (U/mL) was measured in 50 μL of medium using the uPA Activity Assay Kit (Merck Millipore).

### uPA/uPAR silencing

A sequence targeting the uPA receptor (NM_001005376) was designed using BLOCK-iT RNAi Designer (https://rnaidesigner.invitrogen.com/rnaiexpress) (Life Technologies, Melbourne, Australia). The oligonucleotide sequence:

TGCTGTTCAGAGGAGCATCCATGGGTGTTTTGGCCACTGACTGACACCCATGGGCTCCTCTGAA, and its complement were annealed and ligated into pcDNA-6.2-GW/EmGFP-miR to create pcDNA6.2-uPARKDY (BLOCK-iT Lentiviral Pol II miR RNAi Expression System). pcDNA6.2-uPARKDY was transfected into KHOS cells using Lipofectamine 2000 (Life Technologies) and stable transfectants were selected using blasticidin. Knockdown was confirmed by western blot and qRT-PCR.

### *In vivo* metastasis studies

All animal experimentation was approved by, and carried out in strict accordance with the recommendations of, The University of Queensland Health Sciences Ethics Committee (Approval Numbers: UQDI/PAH/291/12/NHMRC and UQDI/214/13/NHMRC). Four to six female 6-week-old BALB/c nude mice were used in each group and experiments were repeated at least once. Each cell line was injected intra-femorally at a concentration of 5 x 10^6^ cells/mL and injected in a volume of 10 µL (50,000 cells). In experiments involving genetically-modified cell lines animals were injected intra-femorally (i.f.) with KHOS WT, KHOS-KD silenced uPAR) or KHOS-SCR (scrambled shRNA) (2.5 x 10^6^ cells/mL) or vehicle. In studies involving administration of WX-340 (at a dose of 10 mg/kg) mice were injected i.p. 3 times/week, beginning 3 days after i.f. injection. Mice were euthanized when tumours reached 10 mm in diameter. Lungs were collected after perfusion with 4% paraformaldehyde, fixed, sectioned and H&E stained. Metastasis was measured by either visually counting the number of lesions in the entire mouse lung section for each mouse or by quantifying metastatic burden (% area covered by metastatic lesions/% total lung area) in each lung, using Nikon NIS-Elements software (Nikon Corporation Instruments, Tokyo, Japan), at 10X magnification.

## Results

### OS cell lines display differences in metastatic potential

**Fig 1:**
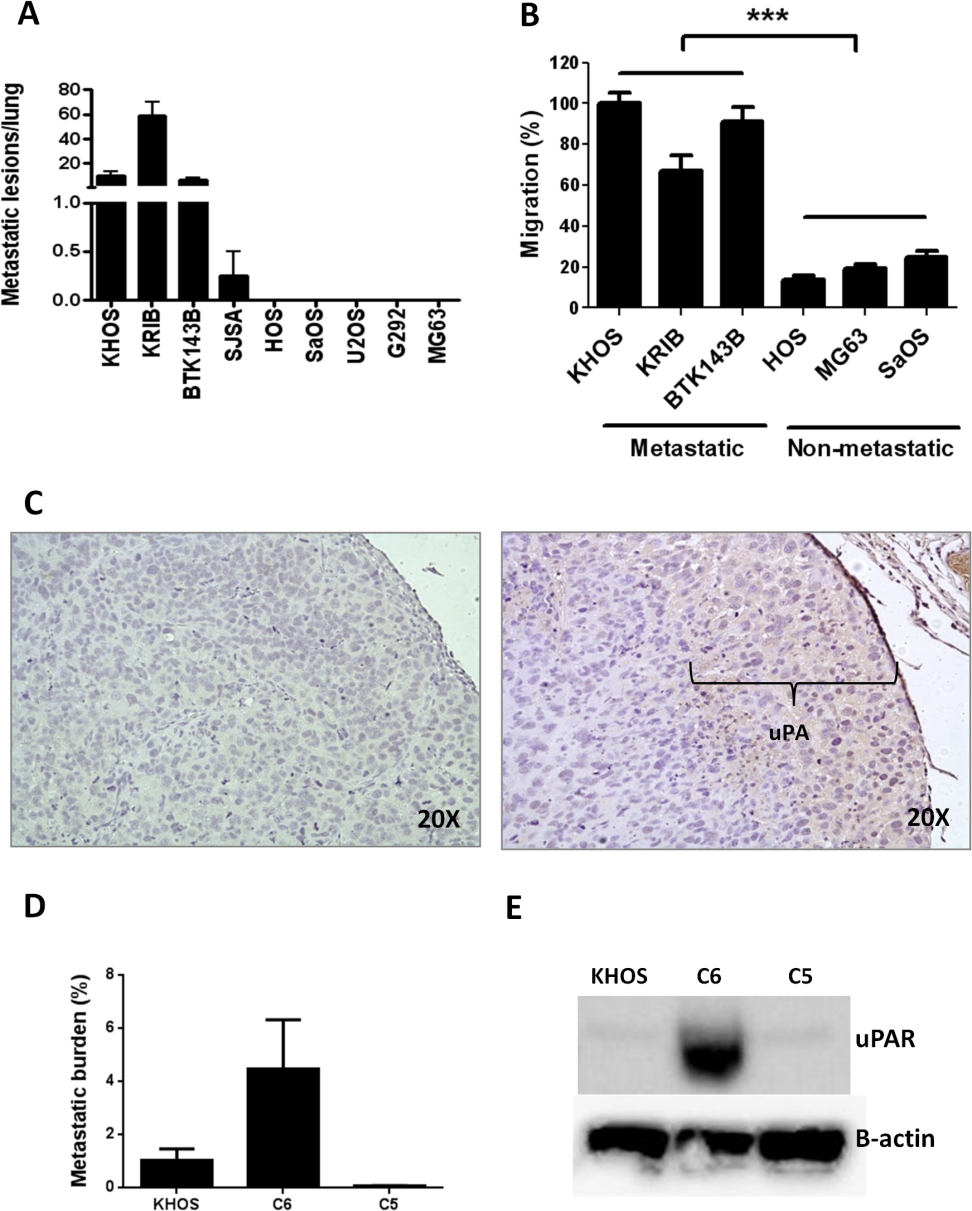
The migratory and metastatic behaviour of OS cells is associated with uPA/uPAR expression. **(A)** Metastatic behaviour (number of metastatic lesions/lung) of various OS cell lines injected intra-femorally into mice (*n* = 12). Injection volume was 10 μL and cells were injected at 5 x 10^6^ cells/mL. Mice were culled when tumours reached 10 mm in diameter. Metastatic cell lines: KHOS, KRIB, BTK143B, SJSA. Non-metastatic cell lines: HOS, SaOS, U2OS, G292, MG63. **(B)** *In vitro* migration of selected metastatic and non-metastatic OS cell lines. Cells (100 μL) were seeded at 2 x 10^5^ cells/mL in serum-free medium into the upper Transwell chamber and allowed to migrate for 24 h towards DMEM/20% FCS in the bottom chamber. Cells that migrated were detected with 8 μM calcein-AM and detached with trypsin-EDTA. Fluorescence was measured at 485 nm (exc) and 520 nM (emm). Percentage migration is normalized against KHOS. Experiments were performed in triplicate at least twice. Bars: SEM. ***P < 0.0001. **(C)** Immunohistochemistry on a representative FFPE section of orthotopic OS tumour showing uPA expression (indicated) at the leading edge of the tumour. Left panel: negative rabbit IgG (Dako). Right panel: rabbit anti-human uPA (H-140) (Santa Cruz Biotechnology). Both antibodies were used at 2 μg/mL. **(D)** *In vivo* metastatic behaviour, as described in (A) of KHOS, and two of its derivatives: C6, a highly metastatic subclone and C5, a poorly metastatic subclone (*n* = 4). **(E)** Immunoblotting of protein whole cell extracts showing that high expression of uPAR correlates with an increase in metastatic potential in the C6 subclone compared to the metastatic parental cell line (KHOS) and the poorly metastatic subclone (C5). Mouse monoclonal anti-human B-actin (C4) (Santa Cruz), 1:5000; monoclonal mouse anti-human uPAR (clone 109801) (R&D Systems), 1:500, under non-reducing conditions.

In an earlier study, we showed that the metastatic potential of human OS could be predicted at the time of biopsy by a specific gene signature and by the numbers of osteoclasts present in the primary tumour [8]. In order to identify other factors that contribute to metastatic potential, we screened a suite of human OS cell lines to define their metastatic activity. Mice were injected i.f. with OS cells and tumour growth and the development of lung metastasis was followed for 4–6 weeks. All mice were culled when tumours reached 10 mm in diameter. Four of the nine (44%) cell lines (KHOS, KRIB, BTK143B, SJSA) spontaneously formed pulmonary metastases within 4 weeks of injection, while five (55%) cell lines (HOS, SaOS, U2OS, G292, MG63) did not (Fig 1A). Since cell migration is one of the first steps in the metastatic process [26], we performed *in vitro* migration assays with three representative OS cell lines from each group chosen according to their consistent metastatic and non-metastatic behaviour *in vivo*. We found that migration assays reflected metastatic behaviour *in vivo*, with metastatic cell lines exhibiting significantly (P < 0.0001) more migration than non-metastatic cell lines (Fig 1B).

### The uPA/uPAR system is associated with metastatic behaviour in OS

To identify molecules that may be uniquely associated with metastatic behaviour, we performed an unbiased comparative gene expression analysis comparing two metastatic/migratory OS cell lines (KHOS, KRIB) and two non-metastatic/non-migratory OS cell lines (HOS, U2OS). The analysis identified 134 genes exclusively overexpressed by metastatic OS (FC >2.0, B > 3.0). Gene enrichment showed involvement in cell growth and proliferation (63%), cell death (54%), cellular development (54%), cellular movement (43%) and cell-to-cell signaling (35%). When sorted according to fold change, the most highly differentially expressed gene was uPA (FC = 8.64, B = 17.14) (Table 1A, S1 Table).

**Table 1:** Top hits from (A) transcriptomic, (B, D) proteomic and (C) secretomic analyses.

**A. Transcriptomic analysis (Illumina HumanHT-12 Expression Bead Chip)** of metastatic *vs*. non-metastatic OS cells.				
**Gene**	**Name**	**Accession No.**	**FC**	**B value**
uPA	Urokinase plasminogen activator	NM_002658	8.64	17.14
AHNAK2	AHNAK nucleoprotein 2	NM_024064	7.08	21.51
MLPH	Melanophilin	NM_024101	7.08	21.51
IGFPB4	Insulin-like growth factor binding protein 4	NM_001552	6.14	15.97
NFKBIA	Nuclear factor of kappa light polypeptide gene enhancer in B-cells inhibitor, alpha	NM_020529	6.09	11.48
TMEM158	Transmembrane protein 158	NM_015444	6.00	17.33
VANGL2	Vang-like 2 (van gogh, Drosophila)	NM_020335	5.86	8.84
TNFRSF21	Tumour necrosis factor receptor superfamily, member 21	NM_014452	5.50	13.78
IL11	Interleukin 11	NM_000641	5.48	9.81
C13orf15	Chromosome 13 open reading frame 15	NM_014059	5.40	18.61
**B. Proteomic analysis (SILAC labeling with heavy** ^**13**^ **C**_**6**_^**15**^ **N**_**2**_ **-Lys (metastatic OS cells) and light** ^**13**^ **C**_**6**_^**15**^ **N**_**4**_ **-Ar (non-metastatic OS cells))** plasma membrane proteins				
**Protein**	**Name**	**Accession No.**	**Heavy:Light Ratio**	
uPAR	Urokinase plasminogen activator surface receptor	Q03405	100.36	
NUTF2	Nuclear transport factor 2	P61970	31.17	
DAD1	Defender against cell death	P61803	28.76	
SLCO4A1	Solute carrier organic anion transporter family member 4A1	Q96BD0	24.13	
EPDR1	Mammalian ependymin-related protein 1	Q9UM22	23.48	
DHRS7B	Dehydrogenase/reductase SDR family member 7B	Q6IAN0	14.10	
CDCP1	CUB domain-containing protein 1	Q9H5V8	10.16	
NRP1	Neuropilin-1	O14786	9.35	
SERPINB6	Serpin B6	P35237	7.13	
SPRY4	Protein sprouty homolog 4	Q9C004	6.52	
**C. Secretomic analysis (RayBio Biotin Label-based Human Antibody Array 1)** of conditioned medium from metastatic *vs*. non-metastatic cells.				
**Protein**	**Name**	**Accession No.**	**Exclusive secretion by metastatic cells(units x 10** ^**6**^ **)**	
uPA	Urokinase-type plasminogen activator	P00749	48.96	
VEGF	Vascular endothelial growth factor A	P15692	42.05	
MMP1	Matrix metalloproteinase-1	P03956	38.44	
**D. Proteomic analysis (SILAC labeling as in B) of secreted proteins in conditioned medium from metastatic *vs*. non-metastatic OS cells**				
**Protein**	**Name**	**Accession No.**	**Heavy:Light Ratio**	
**BZW1**	Basic leucine zipper and W2 domain-containing protein 1	Q7L1Q6	14.78	
**PRSS23**	Serine protease 23	O95084	13.35	
**TGFBI**	Transforming growth factor-beta-induced protein ig-h3	Q15582	11.13	
**uPA**	Urokinase-type plasminogen activator	P00749	8.02	
**IGFBP4**	Insulin-like growth factor-binding protein 4	P22692	7.68	
**MMP1**	Interstitial collagenase	P03956	7.27	
**PLTP**	Phospholipid transfer protein	P55058	6.75	
**PTX3**	Pentraxin-related protein PTX3	P26022	6.30	
**UCHL1**	Ubiquitin carboxyl-terminal hydrolase isozyme L1	P09936	6.24	
**S100A4**	Protein S100-A4	P26447	5.34	

To confirm that uPA gene overexpression correlated with secreted uPA protein levels, we performed a cytokine antibody array on conditioned medium collected from cell cultures of the OS cells used for transcriptomic analysis. uPA was the most abundant protein (48.96 arbitrary units) detected in the medium (Table 1C). Furthermore, proteomic analysis of the conditioned medium from SILAC-labelled cultures of metastatic and non-metastatic cells also confirmed uPA as a secreted protein, with a heavy to light ratio (H:L) = 8.02 (Table 1D and S4 Table). Secretion of uPA was further validated by Western blot (S1A Fig). uPA can exist as a precursor high molecular weight protein (HMW-uPA, 55 kDa), or cleaved into two low molecular weight forms: the proteolytically-active LMW-uPA (33 kDA), which was not detectable by our antibody, and the amino terminal fragment (ATF, 18 kDA), which is not proteolytically active but can bind uPAR. Only metastatic cells secreted uPA in both forms (S1 Fig). In addition, measurement of uPA activity in the metastatic cell conditioned medium yielded approximately 200 units/mL, confirming that uPA is secreted in an active form. Finally, uPA expression was confirmed by immunohistochemistry of KHOS tumours established orthotopically in mice (Fig 1C).

To complement our transcriptomic and secretomic profiling, we performed proteomic analysis using SILAC to identify proteins which may be abundantly expressed on the plasma membrane of metastatic OS cells compared to non-metastatic OS cells. An average of 838 proteins were identified in each of three independent runs (S5 Table). Enrichment analysis of the protein list identified involvement in small molecule biochemistry (35%), cell death (26%), cellular movement (22%) and oxidative phosphorylation (11%). After further filtering, the analysis identified 46 plasma membrane-associated proteins with H:L > 2. The most abundantly expressed protein in the metastatic OS cells was the uPA receptor (H:L = 100.36) (Table 1B, S2 Table). To validate the expression of uPAR in OS, we performed a western blot with three highly metastatic OS cell lines (KHOS, KRIB, BTK143B), a poorly metastatic cell line (SJSA) and three non-metastatic cell lines (HOS, U2OS, G292) (S1B and S1C Fig). Expression of uPAR protein was higher in the highly metastatic cells and was associated with metastatic potential. The latter was independent of the expression of Ras in the K-Ras transformed cell lines, KHOS, KRIB and BTK143B (S1B Fig), suggesting that the metastatic potential of OS is associated with uPAR expression and not Ras expression. To further address the possibility that differences in uPA/uPAR may reflect activated Ras signaling rather than a primary causative factor in the progression to a metastatic phenotype, we validated uPAR protein expression in a metastatic parental line (KHOS), in its highly metastatic derivative/subclone (C6), and in its poorly metastatic derivative/subclone (C5) (Fig 1D). The highly metastatic derivative/subclone (C6) displayed very high protein levels of uPAR in comparison with its metastatic parental cell line (KHOS) or the poorly metastatic subclone (C5) (Fig 1E).

The combined transcriptomic, proteomic and secretomic data showed that elevated expression of uPA mRNA, abundant secretion of active uPA protein, and abundant plasma membrane expression of uPAR is restricted to metastatic OS cells. In addition, high levels of uPAR expression are associated exclusively with metastatic OS cell lines, are independent of Ras status, and are increased in a highly metastatic derivative of KHOS. These data suggest that activation of the uPA-uPAR axis could be involved in metastatic conversion and that it may be a major driver of metastatic behavior.

### uPA and uPAR contribute to the regulation of OS metastasis *via* an autocrine loop

To functionally validate the role of uPA/uPAR in the regulation of OS metastasis and to understand the mechanism whereby uPA/uPAR regulates OS metastasis, we investigated the role of uPA/uPAR on cell migration *in vitro*. We had previously shown that *in vitro* migration correlates with metastatic behaviour *in vivo* (Fig 1).

**Fig 2:**
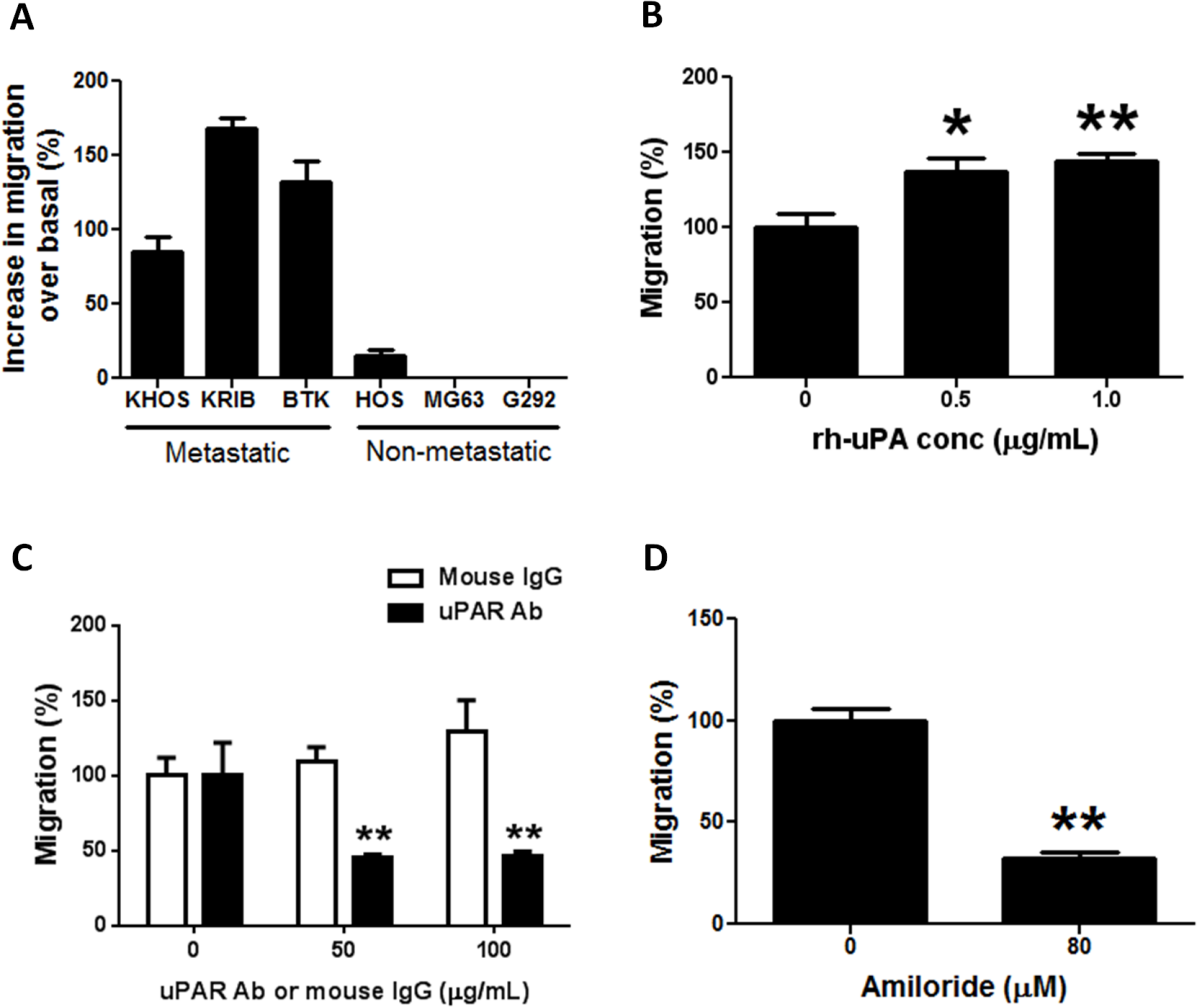
The uPA/uPAR system regulates OS migration. **(A)** Increase in migration over basal levels of metastatic and non-metastatic OS cells in the presence of uPA-rich (200 U/mL) conditioned medium collected from metastatic KHOS cells after 24 h culture. Bars: SEM. **(B)** Increase in migration of metastatic KHOS cells in the presence of 0.5 and 1.0 μg/mL of rh-uPA (R&D Systems). Bars: SEM. *P < 0.02, **P < 0.002. **(C)** Decrease in migration of metastatic KHOS cells in the presence of a neutralizing mAb against uPAR (American Diagnostica). Control: LEAF Purified Mouse IgG (BioLegend). Bars: SEM. **P < 0.005. **(D)** Decrease in migration of metastatic KHOS cells in the presence of a non-toxic concentration (80 μM) of the uPA inhibitor, amiloride. Bars: SEM. **P = 0.0014. Experiments were performed in triplicate at least twice.

Migration assays with metastatic and non-metastatic OS cells were performed with normal growth medium (control) or with metastatic OS cell conditioned medium that constitutively contains approximately 200 U/mL of OS-secreted uPA. A large increase in migration (71.3–181.5%) over basal levels was observed only in metastatic OS cells (Fig 2A). The increase in migration for metastatic cells (e.g. KHOS) in the presence of OS-secreted uPA (71.3–103.9%) was comparable to that observed with exogenously added recombinant human uPA (rh-uPA) (99.0–166.2%) (Fig 2B). Increases in migration over 24 h were not due to cell proliferation (S2 Fig). Furthermore, blocking the uPA receptor with a neutralizing monoclonal antibody significantly (P < 0.005) decreased the inherent ability of metastatic cells (KHOS, KRIB) to migrate (Fig 2C; S3A Fig). In addition, migration assays with a selective inhibitor of uPA, amiloride [27, 28], inhibited KHOS migration (*P* < 0.002) (Fig 2D). This inhibition was not due to amiloride-induced cytotoxicity (S3B Fig).

**Fig 3:**
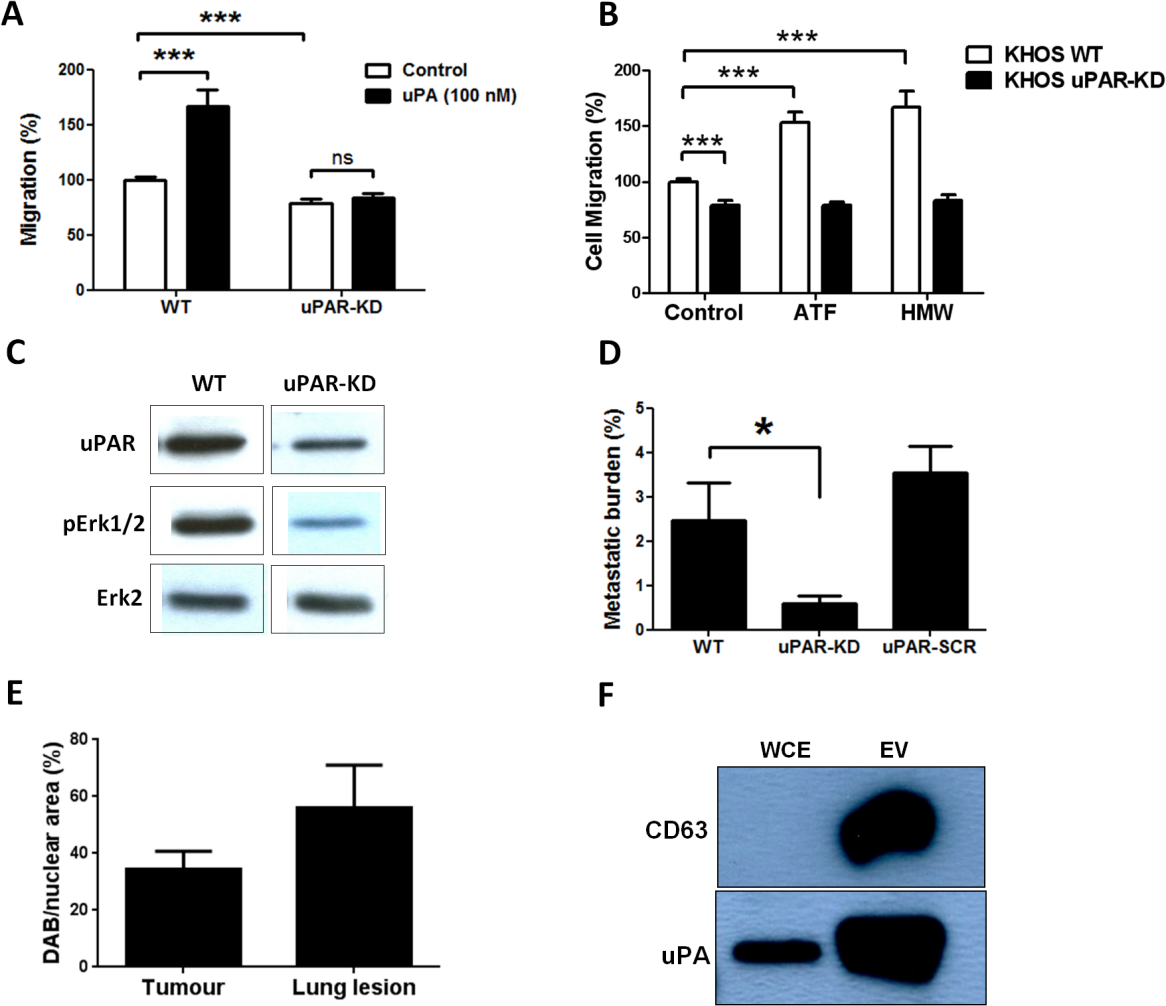
uPAR silencing inhibits migration and metastasis of OS. **(A)** Migration of KHOS WT and KHOS uPAR-KD in the absence (control) or presence of 100 nM uPA (5.4 μg/mL). Percentage migration is normalized against KHOS. Bars: SEM. ***P < 0.0002. **(B)** Migration of KHOS wild-type and uPAR-KD cells in the presence of growth medium (control), 100 nM uPA amino terminal fragment (ATF) or high molecular weight (HMW) uPA. Percentage migration is normalized against KHOS. Migration experiments were performed in triplicate at least twice. Bars: SEM. ***P ≤ 0.0002. **(C)** Reduction of Erk1/2 phosphorylation in uPAR-KD cells but not in WT after 24 h treatment with 100 nM rh-uPA. **(D)** Metastatic burden (% area of lung covered by metastatic lesions/% total lung area) in mice (*n* = 5) injected with KHOS WT, uPAR-KD or uPAR-SCR cells. *P = 0.013. Bars: SEM. **(E)** Quantitative image analysis of DAB-stained FFPE sections of KHOS WT primary tumour and the corresponding lungs after immunohistochemistry with goat anti-human uPAR (Santa Cruz), 1:100. **(F)** Western blot showing expression of CD63 in KHOS-secreted extracellular vesicles (EV) but not in KHOS whole cell extract (WCE), and expression of uPA in both EV and WCE.

Silencing uPAR expression in a functionally representative metastatic cell line, KHOS, using shRNA (uPAR-KD) resulted in approximately 60% uPAR knockdown (S3C Fig), and significantly (P < 0.0002) reduced basal levels of migration (Fig 3A). Whereas, in the presence of rh-uPA, KHOS wild-type cells (WT) displayed a significant (P < 0.0001) increase in migration, uPAR-KD cells failed to respond and did not show an increase in migration (Fig 3A). It is noteworthy that uPAR-KD significantly reduced (P = 0.0002) basal levels of migration (Fig 3A), suggesting that the presence of the uPA receptor contributes to the inherent migratory potential of OS cells. The incomplete inhibition of basal levels of migration in uPAR-KD cells could be attributed to incomplete silencing of uPAR and/or alternate pathways of migration control. The uPAR silencing and neutralising Ab experiments show that maximal concentrations of the neutralizing Ab resulted in a 50% reduction in migration, suggesting that the uPA/uPAR axis contributes 50% to total OS cell migration. Combined, these data show that the ability to produce and/or respond to uPA is restricted to metastatic OS cells. In addition, these data show that uPA acts through the uPAR in an autocrine fashion in metastatic OS cells.

uPA/uPAR-dependent activities could be attributable to proteolytic and/or signalling activities of uPA/uPAR. Therefore, we examined whether uPA-induced migration was regulated by proteolytic or signalling-dependent uPA activities, or both. The uPA ATF contains the uPAR-binding epitope but has no proteolytic activity and hence can be used to probe for uPAR signalling-dependent activity. HMW uPA contains both the proteolytic LMW fragment as well as the ATF. KHOS WT cells, which have an intact uPA receptor, increased migration (P < 0.0001) in response to both the ATF and HMW fragments, whilst uPAR-KD cells did not respond to either uPA form (Fig 3B). These data show that uPAR-dependent *in vitro* migration can be mediated *via* uPA-dependent signalling events and is, in part, independent of uPA proteolytic activity. MAPK signalling has been shown to be activated *via* the uPA/uPAR axis [29]. Consistent with this, we now show that silencing of the uPAR receptor leads to reduced MAPK signalling as evidenced by a reduction in phospho-Erk1/2 in uPAR-KD but not in KHOS WT cells, after treatment with uPA (Fig 3C).

Given that uPAR silencing inhibited OS cell migration *in vitro*, we hypothesized that uPAR silencing would also reduce OS metastasis *in vivo*. Mice were injected i.f. with KHOS WT, KHOS-KD or uPAR-SCR (scrambled). Lung metastasis was significantly (*P* < 0.02) reduced in the animals bearing uPAR-KD tumours compared to WT or uPAR-SCR (Fig 3D). The reduction in uPAR expression in the KHOS KD tumours was confirmed by immunohistochemistry (S4 Fig). Similar to the reduction in migration, the absence of complete inhibition of metastatic burden may be due to incomplete uPAR silencing. More importantly, a 60–70% reduction in uPAR expression (S3C and S4 Figs) is sufficient to cause significant inhibition of pulmonary metastasis. While a significant difference *(*P < 0.02) was observed in metastatic potential in the absence of the uPA receptor, no difference was observed in tumour growth (S3F Fig), indicating that the uPA/uPAR system regulates OS metastasis independent of tumour growth. Further validation for the role of uPA/uPAR in promoting pulmonary metastasis is provided by quantitative image analysis of KHOS WT primary tumours and corresponding lung lesions which shows a 63.8% increase in uPAR expression in the metastatic lesions (Fig 3E and S5 Fig).

### Metastatic OS cells secrete uPA in soluble and in EV-bound form

Extracellular vesicles (EVs), including exosomes, are secreted into the microenvironment by a number of cancer cells and mediate tumour progression and metastasis through the intercellular transfer of RNA and signaling proteins [30–33]. To date, there are no publications describing the existence or secretion of EVs by OS cells. Thus, we investigated whether metastatic OS cells (KHOS) could secrete EVs and whether these vesicles may contain biologically active uPA as part of their cargo. EV isolation was performed by repeated sequential centrifugation of 24 h KHOS conditioned medium. The resulting pellet yielded 0.91 μg/mL of total protein, showed particles in the expected range of 30–90 nm for exosome diameter, and positive staining for CD63 protein, a commonly used exosome marker [34, 35] (Fig 3F). Measurement of uPA activity yielded 122 U/mL in the exosome pellet and 260 U/mL in the exosome-free supernatant. uPA protein expression was present in KHOS whole cell extracts as well as abundant in EVs (Fig 3F). Our data demonstrate that uPA is secreted by metastatic OS cells in an active soluble form as well as in an active exosome-bound form.

### The bone marrow microenvironment contributes to OS cell migration through uPA/uPAR

**Fig 4:**
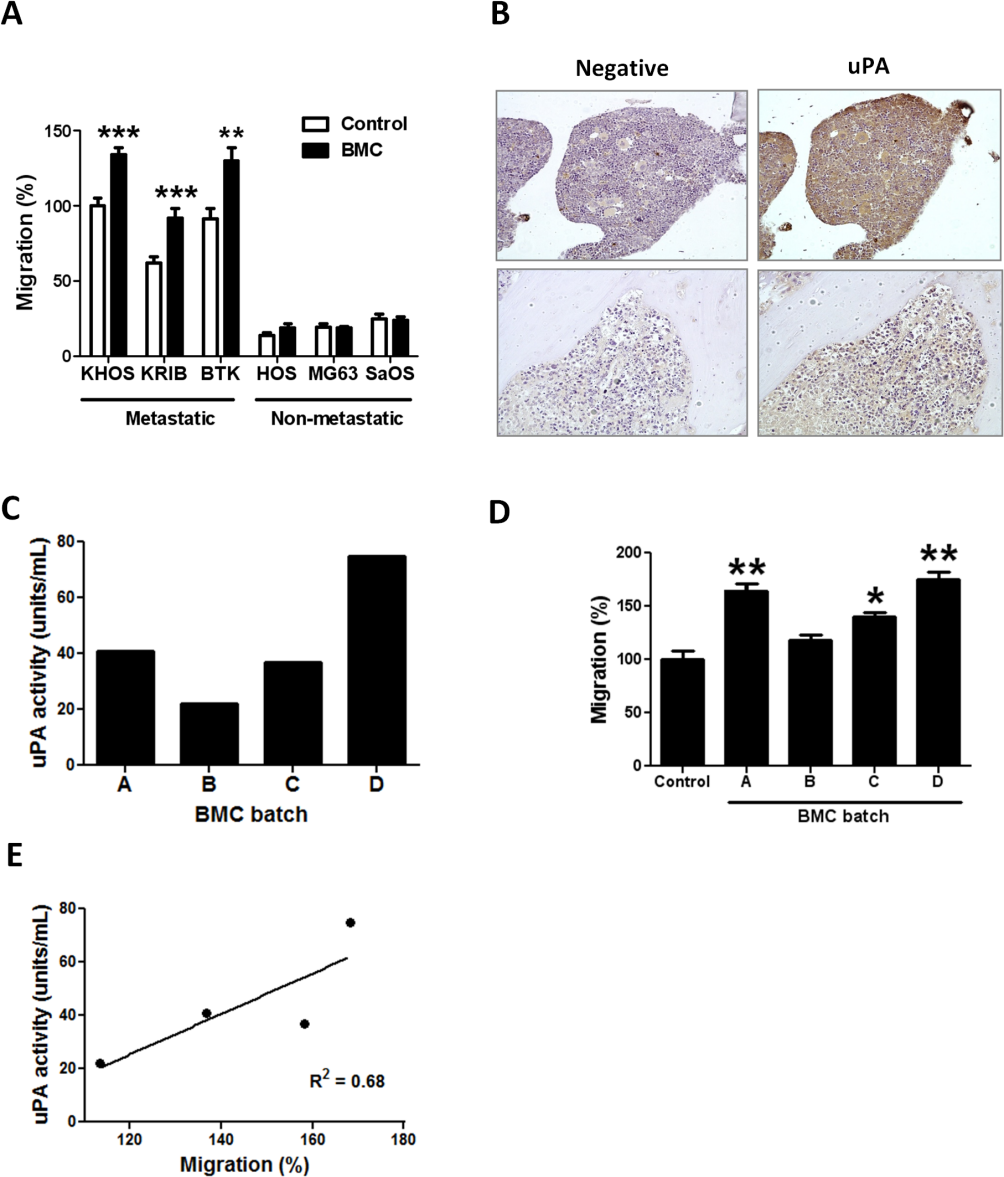
Bone marrow increases the migration of metastatic OS through uPA/uPAR. **(A)** Migration of metastatic and non-metastatic OS cell lines in the presence of uPA-rich medium (20–80 U/mL) collected from bone marrow cell cultures (BMC). Percentage migration is normalized against KHOS control. Bars: SEM. ***P <0.0001, **P = 0.0084. Experiments were performed in triplicate at least twice. **(B)** Immunohistochemistry of the same FFPE section of mouse bone showing expression of uPA in bone marrow cells (right panels). Left panels: negative rabbit IgG (Dako), 2 μg/mL. Right panels: rabbit anti-human uPA (H-140) (Santa Cruz Biotechnology), 1:100 (2 μg/mL). The top panels show strong uPA staining in an area in the proximal epiphysis near the epiphyseal line, an area of rapid bone turnover, rich in osteoblasts and osteoclasts which secrete uPA. The bottom panels show and area in the diaphysis in the border of the medullary cavity and compact bone, where osteoblasts and osteoclasts are not so abundant. Magnification: 10X. **(C)** Activity of uPA in various batches (A-D) of bone marrow conditioned medium measured by a uPA Activity Assay Kit (Merck Millipore). **(D)** Migration of KHOS cells in the presence of normal growth medium (control) and each of the batches (A-D) of bone marrow conditioned medium in (C). Percentage migration is normalized against KHOS control. Bars: SEM. *P < 0.02, **P < 0.009. Experiments were performed in triplicate at least twice. **(E)** Linear regression of uPA activity *vs*. migration. Slope = 0.7589 ± 0.3693; Y-intercept when X = 0.0 is -65.61 ± -53.78; X-intercept when Y = 0.0 is 86.45.

We had previously shown that conditioned medium collected from cultures of bone marrow cells (BMC) significantly (P < 0.0001) increased the migration of metastatic KHOS cells but not that of non-metastatic HOS cells [8]. Given that the uPA/uPAR system determines inherent metastatic potential in OS, we investigated whether stromal cells in the bone microenvironment could also contribute to OS migration through the uPA/uPAR axis. Firstly, we extended our initial observations to include a larger number of metastatic and non-metastatic OS cells, and confirmed that BMC conditioned medium significantly (P < 0.008) increases the migration of a number of metastatic OS cells but not that of non-metastatic cells (Fig 4A). We then confirmed the expression of uPA in BMC by immunohistochemistry of mouse bone sections (Fig 4B).

To investigate the effect of BMC on OS gene expression, we grew metastatic OS cells (KHOS, KRIB) for 24 h in the presence of normal growth medium or BMC conditioned medium which contained 70 U/mL of BMC-derived uPA. A transcriptomic analysis was performed to compare the upregulation of genes between BMC-treated and BMC-untreated OS cells. The analysis identified 208 genes which were exclusively overexpressed by metastatic OS cells (FC >2.0, B > 3.0). uPA was the most significantly (B = 18.82, FC = 7.97) upregulated gene in metastatic OS cells after exposure to BMC (S3 Table). In addition, uPAR was also significantly upregulated (B = 6.93, FC = 3.43) by BMC (S3 Table). These results were confirmed by a western blot of metastatic OS cells showing an average 48% increase in uPAR expression after 24 h treatment with HMW uPA (S3D Fig). The combined results indicate that BMC secrete uPA which acts in a paracrine fashion to induce uPA and uPAR mRNA expression. It is also possible that BMC may secrete other, yet to be identified factor(s) that induce uPA or uPAR mRNA expression in OS cells.

To investigate whether the combined upregulation of uPA mRNA and BMC-secreted uPA could impact metastatic OS cell migration we used several batches of BMC conditioned medium with varying levels of uPA (22–75 units/mL) in migration assays (Fig 4C). All batches of conditioned medium increased migration of KHOS cells by 25–80%, but significantly (*P* < 0.009) greater migration was observed with batches that had higher uPA activity (Fig 4D). A uPA activity of 40–80 U/mL in bone marrow conditioned medium increased migration in a comparable manner to that induced by 1 μg/mL of rh-uPA (Fig 2B). A significant correlation between uPA activity in the BMC conditioned medium and migration was confirmed by Pearson correlation (r = 0.88) (not shown) and linear regression (R^2^ = 0.68) (Fig 4E). To demonstrate that human OS cell uPAR was responding to the binding of mouse BMC uPA, we performed a migration assay of KHOS cells in the presence of rh-uPA and recombinant mouse uPA. Both sources of uPA significantly (*P* < 0.05) increased the migration of KHOS cells (S3E Fig).

These findings indicate that stromal cells in the bone marrow microenvironment secrete uPA which binds directly to the uPAR expressed on metastatic OS cells to further stimulate uPA-dependent OS cell migration/metastasis and uPA/uPAR expression/secretion. Thus, uPA operates *via* paracrine and autocrine loops in the bone marrow compartment to induce OS metastasis and migration. Moreover, these data also indicate that the selective overexpression of uPAR by OS cells is a major driver of the conversion of a non-metastatic OS cell to a metastatic OS cell.

### Pharmacological inhibition of uPA decreases OS metastasis *in vivo*

**Fig 5:**
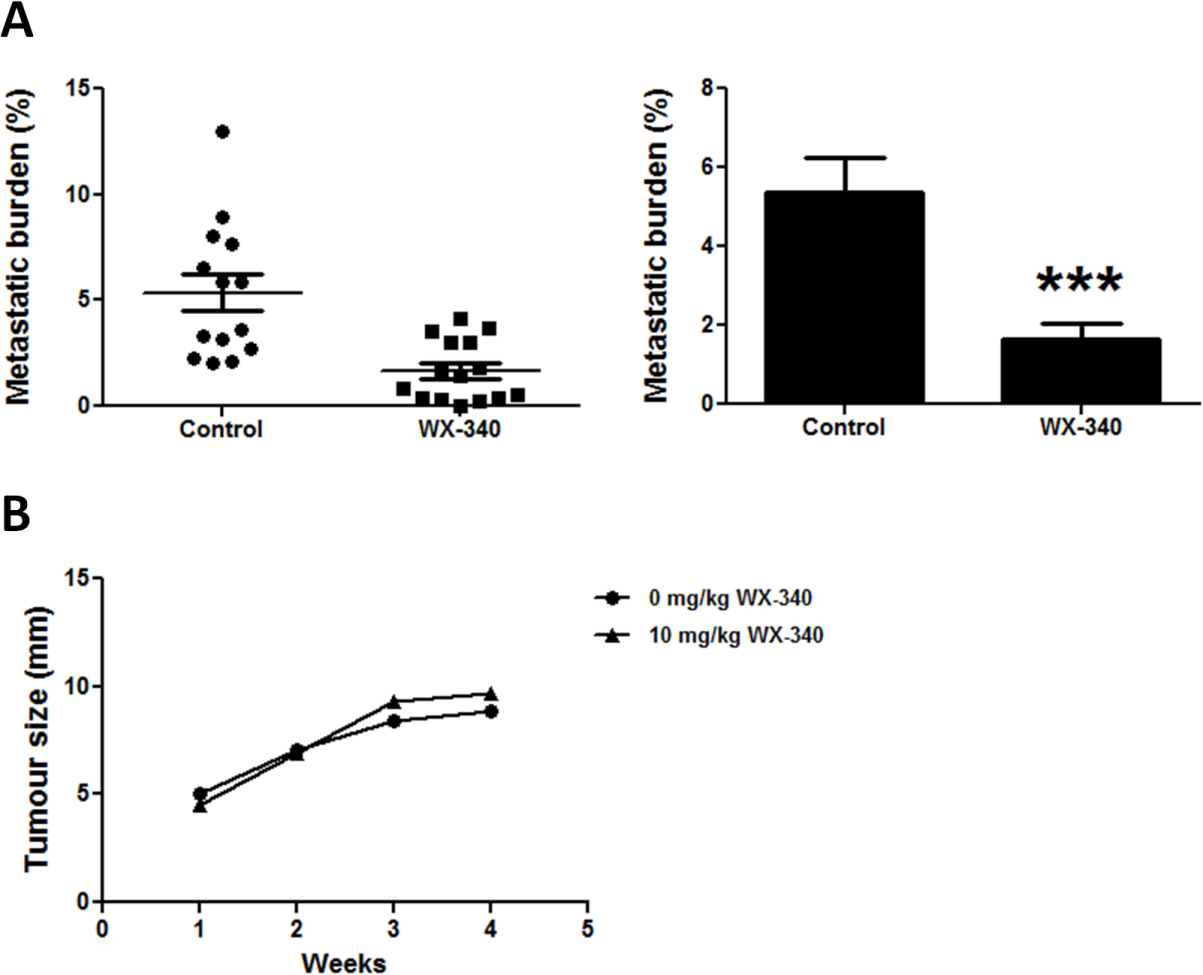
Inhibition of OS metastasis by the uPA inhibitor, WX-340. **(A)** Metastatic burden (% area covered by metastatic lesions/% total lung area) in animals treated with vehicle (control) (*n* = 14) or treated with 10 mg/kg WX-340 (*n* = 15) i.p. 3 times/week. Left: scatter plot. Right: Bars: SEM. ***P = 0.0004. **(B)** Average tumour growth of mice in (A).

We used our mouse model of OS metastasis to test whether WX-340, a small molecule, high potency, specific, active site competitive uPA inhibitor could reduce OS metastasis *in vivo*. Treatment of KHOS tumour-bearing mice with WX-340 significantly (*P* = 0.0004) reduced pulmonary metastasis in the experimental animals compared to vehicle controls when administered early, i.e. beginning 3 days after i.f. injection (Fig 5A). When WX340 was administered after the tumours were allowed to first establish for 2 weeks, no inhibition of metastasis was observed (data not shown), indicating that uPA inhibition by WX340 specifically targets the progression of metastatic disease in the early stages of tumour development. Furthermore, there was no difference in tumour size in WX-340 treated and untreated animals over the duration of the experiment (Fig 5B), indicating that the mechanism by which uPA inhibits OS metastasis is independent of tumour growth.

## Discussion

In the present study we make a number of novel observations relating to the mechanism regulating OS metastasis. In particular we show that (i) activation of the uPA/uPAR axis is a fundamental event driving the metastatic behaviour of OS cells, (ii) transition from a non-metastatic to a metastatic phenotype is defined by the induction of an autocrine loop in which metastatic OS cells secrete soluble active uPA that binds and activates the uPAR on their plasma membrane, (iii) metastatic OS cells secrete exosomes and that these contain active uPA, (iv) stromal cells contribute to uPA-dependent metastasis *via* secretion of uPA and activation of the uPA/uPAR axis in OS cells *via* a paracrine loop, v) that the driver of uPA-mediated metastasis is the dysregulated and selective overexpression of uPAR in metastatic OS cells and (vi) uPA-dependent activation of migration can be mediated *via* uPA-dependent signalling independently of proteolytic activity. Finally, and most importantly, we show that a novel pharmacological inhibitor of uPA/uPAR selectively prevents pulmonary metastasis in an orthotopic model of OS metastasis.

**Fig 6:**
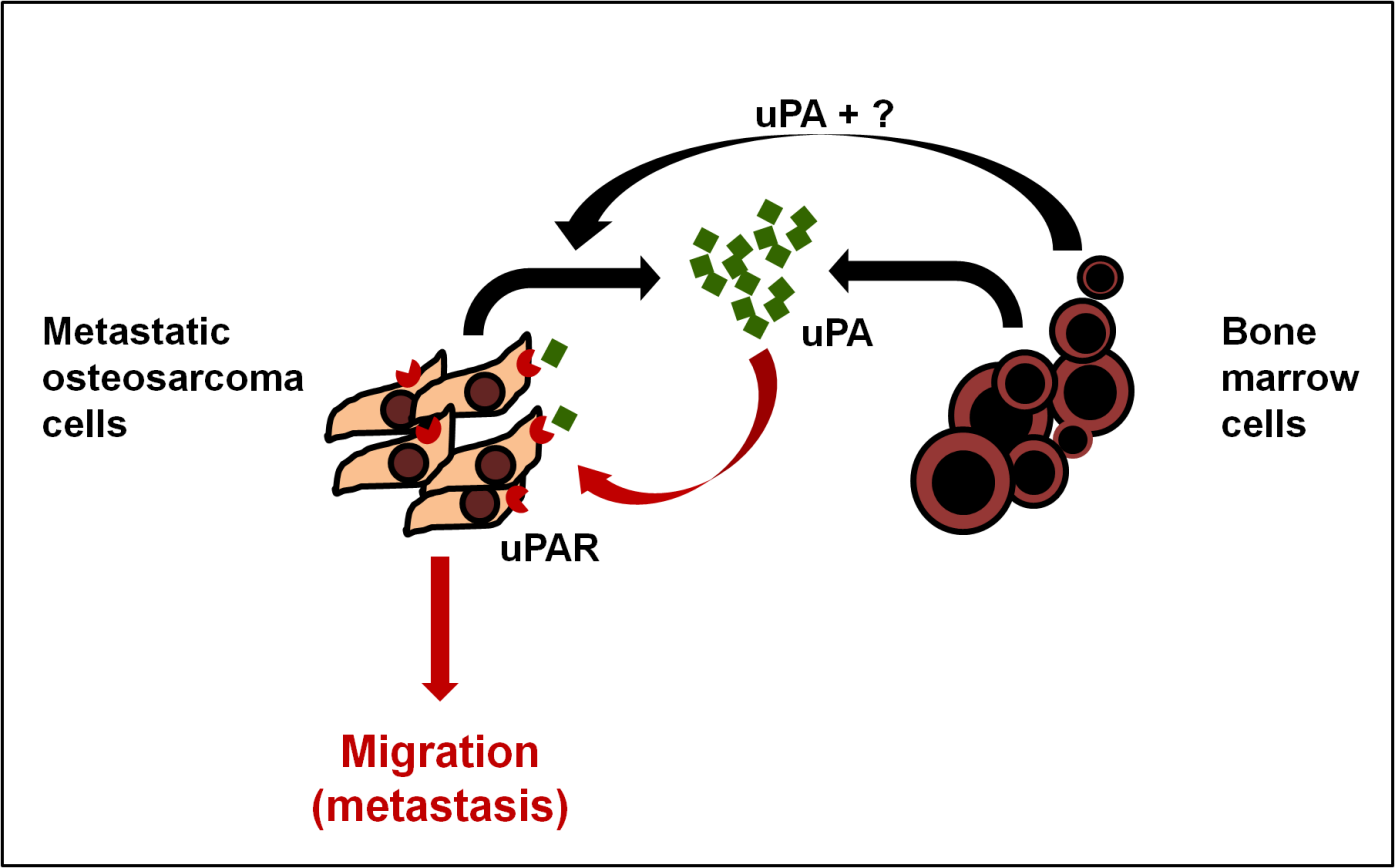
Model of uPA/uPAR signaling in OS and the bone microenvironment. Osteosarcoma cells express high levels of uPA mRNA and secrete uPA into the surrounding microenvironment. They also express abundant uPAR on their cell membranes. The OS-secreted uPA acts in an autocrine fashion by binding to OS uPAR and promoting OS cell migration, invasion and metastasis by activating intracellular signaling. In addition, bone marrow cells in the surrounding microenvironment contribute in a paracrine fashion to both increase uPA/uPAR expression by OS cells, and to the pool of secreted uPA in the microenvironment, to further increase metastasis.

OS patients with pulmonary metastases continue to have survival rates of only 10–20% [2]. The lack of progress in treating metastatic OS likely reflects our poor understanding of the molecular mechanisms regulating the metastatic process. Early studies reported that uPA expression correlated with an invasive or metastatic phenotype in human OS [14–19]. These studies provided initial evidence for the potential importance of the uPA/uPAR axis in OS metastasis, but were limited to a restricted rat cell line model and did not address the mechanism by which uPA/uPAR contributed to metastasis. Using a multi-omics strategy and a cohort of human OS cell lines, we now show that paracrine and autocrine activation of the uPA/uPAR axis is a characteristic feature restricted to metastatic OS cells (Fig 6). Specifically, uPA is exclusively overexpressed and secreted by metastatic OS cells. Moreover, uPAR is selectively upregulated on the plasma membrane of metastatic OS cells only, and its inhibition reduces OS metastasis *in vivo*. These data indicate that the transition from a non-metastatic to a metastatic phenotype is accompanied by the selective activation of an autocrine uPA/uPAR axis in OS cells. In addition to autocrine activation we also show that BMC constitutively express/secrete uPA which has little effect on non-metastatic OS cells (which don’t express uPAR) but further stimulates migration in metastatic OS cells (Fig 6). Given that stromal cells secrete uPA it is clear that the driver of uPA-mediated metastasis is the dysregulated and selective overexpression of uPAR in metastatic OS cells. The molecular/genetic basis for the metastasis-specific activation of the uPA/uPAR axis remains unknown but is clearly heritable and contributes significantly to the acquisition of the metastatic phenotype in OS. For example, pharmacological or genetic inhibition of the uPA/uPAR axis reduces metastatic activity approximately 3-fold highlighting the relative importance of this axis to overall metastatic activity. This is consistent with earlier reports that overexpression of uPA and uPAR are causally associated with tumour metastasis and poor prognosis in a number of other human cancers [11, 36]. Our own *in vivo* data show an increase in uPAR expression in metastatic lesions compared to OS primary tumours. Thus, our data suggest that activation of the uPA-uPAR axis may drive metastatic conversion in OS.

Immunohistochemical analysis of tumours derived from metastatic OS cells (Fig 1C), indicate that the uPA-secreting and uPA-responsive cells are present at the leading edges of the tumour. This is consistent with evidence showing that cells at invasive tumour “fronts” undergo an epithelial mesenchymal transition in response to signals from the microenvironment, migrate into the stroma, and go on to form metastatic lesions at distant sites [37, 38]. The presence of OS cells expressing relatively high levels of uPA/uPAR at the tumour “front” indicates that the metastasising fraction of the tumour is subject to modulation by surrounding cancer cells and the adjacent stroma. In this regards, our data suggest that the bone marrow cells near the tumour front could induce the expression of uPA and uPAR in metastatic OS cells as well as their migration in a paracrine fashion (Fig 6). Other factors secreted by BMC, which which may also contribute to the induction of uPA/uPAR expression in OS, remain to be identified.

Our data showing the existence and secretion of EVs, including CD63-positive exosomes, by metastatic OS cells opens the possibility that in OS, as in other cancers, exosomes may contribute to tumour progression and metastasis through local and distant intercellular communication [39, 40]. For example, exosomes contribute to the establishment of a pre-metastatic niche by enhancing angiogenesis, remodelling stromal cells and by promoting extracellular matrix degradation [31]. Many of these functions can also be mediated by the activation of the uPA/uPAR axis [10–13]. Given that OS-secreted exosomes contain active uPA, it is reasonable to suggest that metastatic OS cells can influence metastatic behaviour *via* locally secreted uPA and at distant sites *via* uPA-containing exosomes.

Whilst identifying the biochemical basis of uPA-induced metastasis was not central to this study, we were able to generate some insight into this process. Although the uPA receptor lacks a transmembrane signalling domain it is able to modulate intracellular signalling *via* binding to G protein-coupled receptors, receptor tyrosine kinases and integrins [41]. For example, the interaction of uPAR with integrins regulates cell adhesion, EGFR-dependent proliferation and the ERK/MAPK pathway, as well as paxillin-associated adhesion and migration [42]. In the present study we show that an uPA/uPAR/MAPK axis exists in metastatic OS cells which can be independent of uPA-dependent proteolytic activity. Significantly, we show that uPA-dependent activation of MAPK is associated with migration and metastasis. This is supported by the observation that migration was induced in response to the proteolytically-inactive amino terminal fragment of uPA, and that Erk1/2 phosphorylation was inhibited by uPA in the uPAR-KD but not in the KHOS WT cells. Whilst uPA-induced migration involves the non-proteolytic functions of uPA, the proteolytic actions of uPA could contribute to other processes involved in overall metastatic activity *in vivo*. For example, the uPA/uPAR system plays a critical role in malignancy and has been shown to contribute to metastasis by degrading the ECM through an extracellular proteolytic cascade which leads to cellular migration [43, 44].

A number of strategies targeting the uPA/uPAR axis have been pursued in a number of cancers [42, 45, 46]. Recently, a small molecular weight, active site competitive serine protease inhibitor, WX-UK1, has been shown to inhibit tumour invasion *in vitro* and in animal models of cervical, breast and head and neck cancers, and to inhibit breast carcinoma cell growth and metastasis [47, 48]. In subsequent Phase I/Ib/IIa clinical trials in cancer patients, WX-UK1 treatment in combination with capecitabine resulted in a clinical benefit with no adverse effects [49]. Mesupron, an oral pro-drug of WX-UK1, is currently in Phase II clinical trials for pancreatic and breast cancer [50, 51]. In this study, we have used another pro-drug of WX-UK1, WX-340, a new small-molecule, high potency, specific, active site competitive uPA inhibitor in the research and preclinical proof-of-concept state. In our orthotopic model of OS metastasis, treatment of animals with WX-340 resulted in a 3.2-fold decrease in pulmonary metastasis compared to vehicle-treated animals. Interestingly, inhibition of uPA and the subsequent decrease in metastasis was independent of effects on tumour growth. These data indicate that uPA/uPAR inhibitors are highly selective for inhibiting metastasis and possess no direct anticancer activity (i.e. no inherent cytostatic or cytotoxic activity).

## Supporting Information

S1 Fig (A) uPA is exclusively secreted by metastatic OS cells. Western blot of serum-free conditioned medium collected from 24 h cultures of metastatic (KHOS, KRIB) and non-metastatic (HOS, U2OS) OS cell lines. Medium was concentrated in 10,000 MWCO ultracentrifugal units (Millipore) and 20 μg of protein loaded per lane. uPA was detected with rabbit antibody to human uPA (Santa Cruz) at 1:2000. The presence of the 18 kDa amino terminal fragment (ATF) is probably due to cleavage of uPA under reducing conditions. Lane 1: KHOS. Lane 2: KRIB. Lane 3: HOS. Lane 4: U2OS. (B) uPAR protein expression is associated with the metastatic potential of OS cell lines and is independent of Ras expression. Western blots of whole cell extracts from metastatic OS cells (KHOS, KRIB, BTK143B, SJSA) and non-metastatic cells (HOS, U2OS, G292). uPAR was detected under non-reducing conditions with monoclonal mouse anti-uPAR (R&D Systems) at 1:500. Ras was detected under reducing conditions with mouse anti-Ras (BD Biosciences) at 1:2000. (C) uPAR protein expression is associated with a metastatic phenotype in OS cell lines. Quantitative analysis of the uPAR data in (B) was performed with Fusion-SL image analysis software (Vilmer Lourmat). uPAR expression was normalized to β-actin.
(PPTX)

S2 Fig Increase in migration in the presence of uPA is not due to cell proliferation. **(A)** KHOS cells were seeded at 5 x 10^4^ cells/mL and a proliferation assay was performed in the presence of 100 nM (5.4 μg/mL) of rh-uPA for 24 h using the CellTiter 96 AQ_ueous_ One Solution Cell Proliferation Assay. **(B)** Proliferation assay was performed as for 2A with metastatic cell lines (KHOS, KRIB, BTK143B) and non-metastatic cell lines (HOS, U2OS, SaOS). Experiments were performed in triplicate at elast twice.
(PPTX)

S3 Fig uPA/uPAR regulates OS migration and metastasis. **(A)** Migration of metastatic KRIB cells in the presence of 0–100 μg/mL of a neutralizing mAb (American Diagnostica) against uPAR. Bars: SEM. Results of at least two experiments in triplicate. **(B)** Toxicity assay of amiloride in KHOS cells. Assay was performed for 24 h at a cell concentration of 5 x 10^4^ cells/mL using the Cell Titer^96^ AQ_ueous_ One Solution Cell Proliferation Assay (Promega). Results of at least two experiments in triplicate. **(C)** Gene expression (PCR) of uPAR in KHOS cells before (WT) and after shRNA silencing (uPAR-KD). **(D)** uPAR expression (immunoblotting) in KHOS, KRIB and BTK143B cells after 24 h treatment with 100 nM HMW uPA. Mouse anti-human uPAR (clone 109801) (Santa Cruz), 1:200; Mouse monoclonal anti-human B-actin (C4) (Santa Cruz), 1:5000. Quantitative analysis was performed to correct for B-actin, the detection of which was affected by the WB non-reducing conditions. **(E)** Migration of KHOS cells in the presence of recombinant human (rh) and recombinant murine (rm) uPA, at 1 μg/mL. Percentage migration is normalized against KHOS control. Results of at least two experiments in triplicate. Bars: SEM. *P < 0.04. **(F)** Tumour growth, measured as tumour diameter (mm), in mice (*n* = 5) injected intra-femorally with KHOS WT, uPAR-KD or uPAR-SCR. Bars: SEM.
(PPTX)

S4 Fig Reduction in uPAR protein expression in KHOS-KD tumours. **(A)** Representative FFPE tumour sections from different mice (a, b, c) injected with KHOS-SCR (control) or KHOS-KD. IHC using a commercial uPAR antibody (Santa Cruz goat anti-human uPAR, 1:200), and DAB staining (*brown*). Magnification: 20X. **(B)** Quantitative image analysis was performed using ImmunoRatio [24]. Bars: SEM.
(PPTX)

S5 Fig uPAR expression in primary KHOS WT tumours and metastatic lesions. Immunohistochemistry in FFPE sections. uPAR antibody (Santa Cruz), 1:200. DAB staining *(brown)*.
(PPTX)

S1 Table Metastatic OS cells exhibit high expression of uPA mRNA. Genes upregulated (FC > 2.0, B value > 3.0) in metastatic OS cells compared to non-metastatic OS cells. Transcriptomic analysis performed on Illumina HT-12 Expression BeadChips.
(PDF)

S2 Table Metastatic OS cells exhibit high abundance of plasma membrane-bound uPAR. Proteins present on the plasma membrane of metastatic OS cells compared to non-metastatic OS cells. Quantitative proteomic analysis was performed using SILAC labelling. Heavy (metastatic OS cells) and light (non-metastatic OS cells) lysates were mixed 1:1 by protein weight and then separated by 10% SDS-PAGE before LC-MS/MS analysis.
(PDF)

S3 Table Bone marrow cells increase uPA mRNA expression in metastatic OS. Upregulated genes (FC > 2.0, B > 3.0) in metastatic OS cells compared to non-metastatic OS cells, after 24 h treatment with bone marrow cell conditioned medium. Transcriptomic analysis performed on Illumina HT-12 Expression BeadChips.
(PDF)

S4 Table Proteomic analysis data of secreted proteins. Serum-reduced medium of 24 h cultures of SILAC-labelled metastatic (KHOS, KRIB) and non-metastatic (HOS, U2OS) OS cells was examined by quantitative proteomic analysis. Three biological replicates were analysed in three independent runs. Analysis shows inverted (i.e. light to heavy) ratios for the comparisons metastatic *vs*. non-metastatic.
(XLSX)

S5 Table Proteomic analysis data of plasma-membrane bound proteins. Plasma membrane proteins of SILAC-labelled metastatic (KHOS, KRIB) and non-metastatic (HOS, U2OS) OS cells were examined by quantitative proteomic analysis. Three biological replicates were analysed in three independent runs. Analysis shows inverted (i.e. light to heavy) ratios for the comparisons metastatic *vs*. non-metastatic comparisons.
(XLSX)
