# Supplementary figures and images for: Progression of Osteosarcoma from a Non-Metastatic to a Metastatic Phenotype Is Causally Associated with Activation of an Autocrine and Paracrine uPA Axis
Source: PLoS One. 2015 Aug 28;10(8):e0133592. doi: 10.1371/journal.pone.0133592 (PMC4552671; doi:10.1371/journal.pone.0133592)

## Slide 1
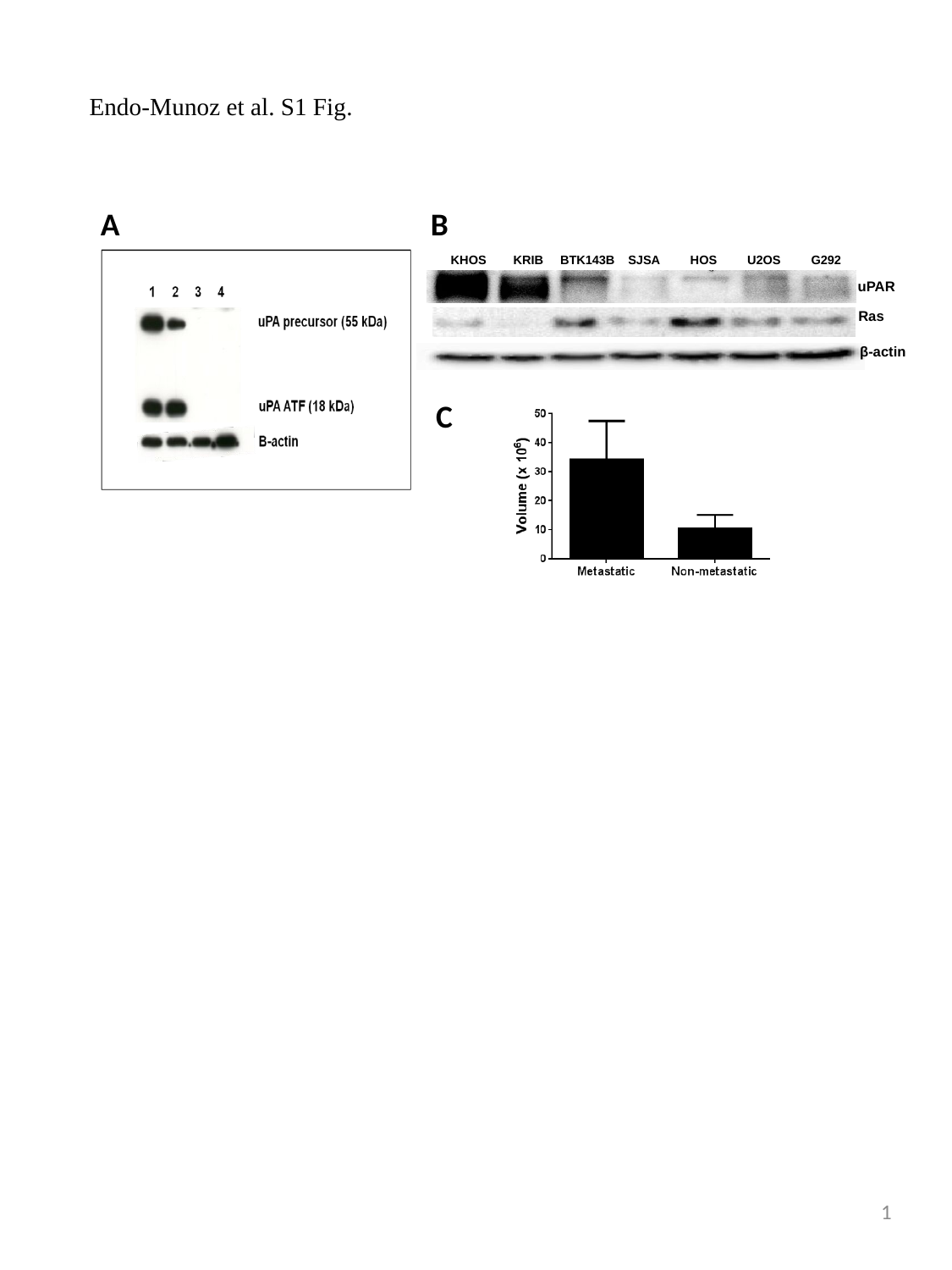

Endo-Munoz et al. S1 Fig.
A
B
KHOS KRIB BTK143B SJSA HOS U2OS G292
uPAR
Ras
β-actin
C
1

Supplement: S1 Fig — Quantitative analysis of the uPAR data in (B) was performed with Fusion-SL image analysis software (Vilmer Lourmat). uPAR expression was normalized to β-actin. (PPTX) [file pone.0133592.s001.pptx]

## Slide 1
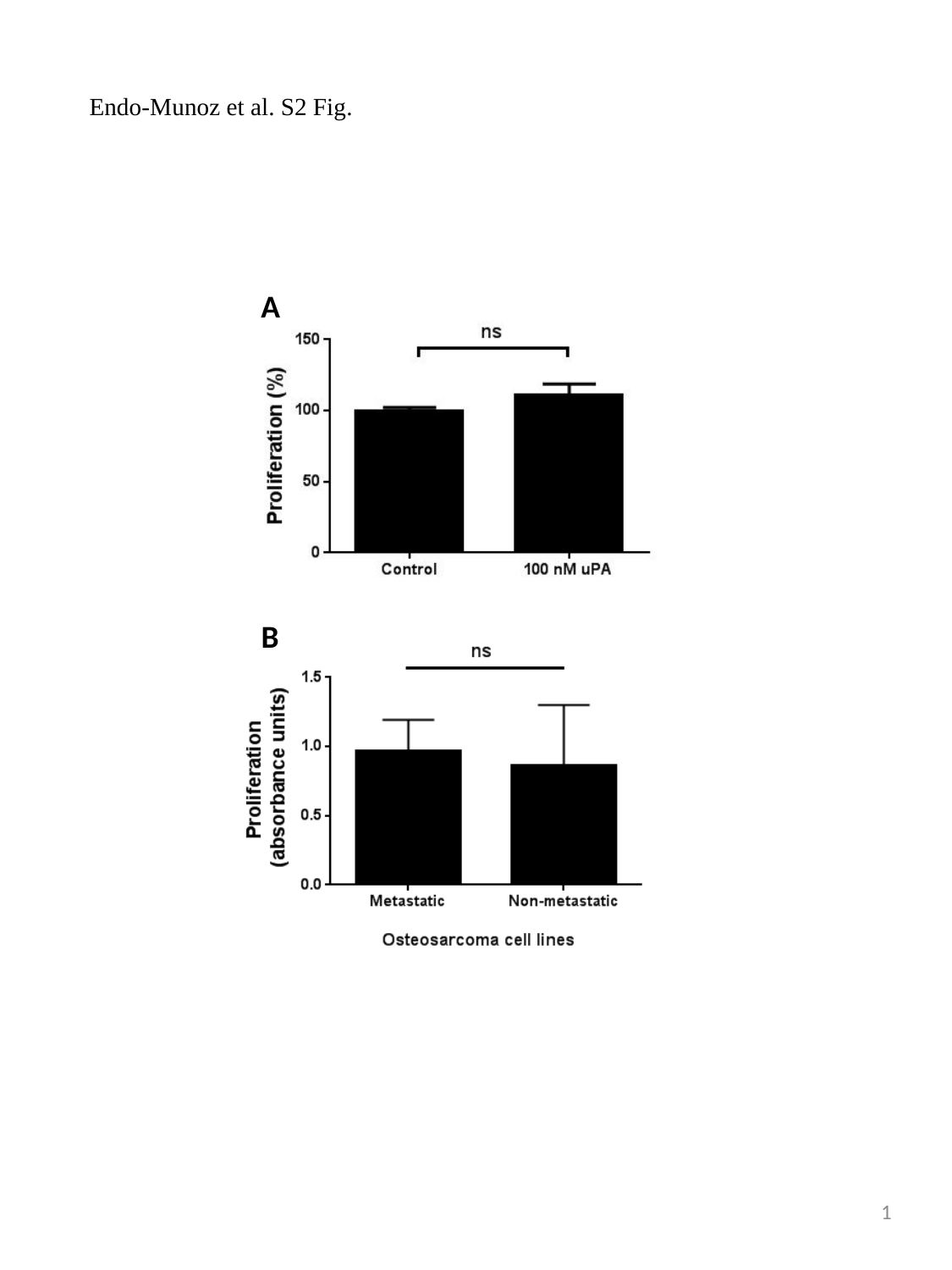

Endo-Munoz et al. S2 Fig.
A
B
1

Supplement: S2 Fig — (A) KHOS cells were seeded at 5 x 10^4^ cells/mL and a proliferation assay was performed in the presence of 100 nM (5.4 μg/mL) of rh-uPA for 24 h using the CellTiter 96 AQueous One Solution Cell Proliferation Assay. (B) Proliferation assay was performed as for 2A with metastatic cell lines (KHOS, KRIB, BTK143B) and non-metastatic cell lines (HOS, U2OS, SaOS). Experiments were performed in triplicate at elast twice. (PPTX) [file pone.0133592.s002.pptx]

## Slide 1
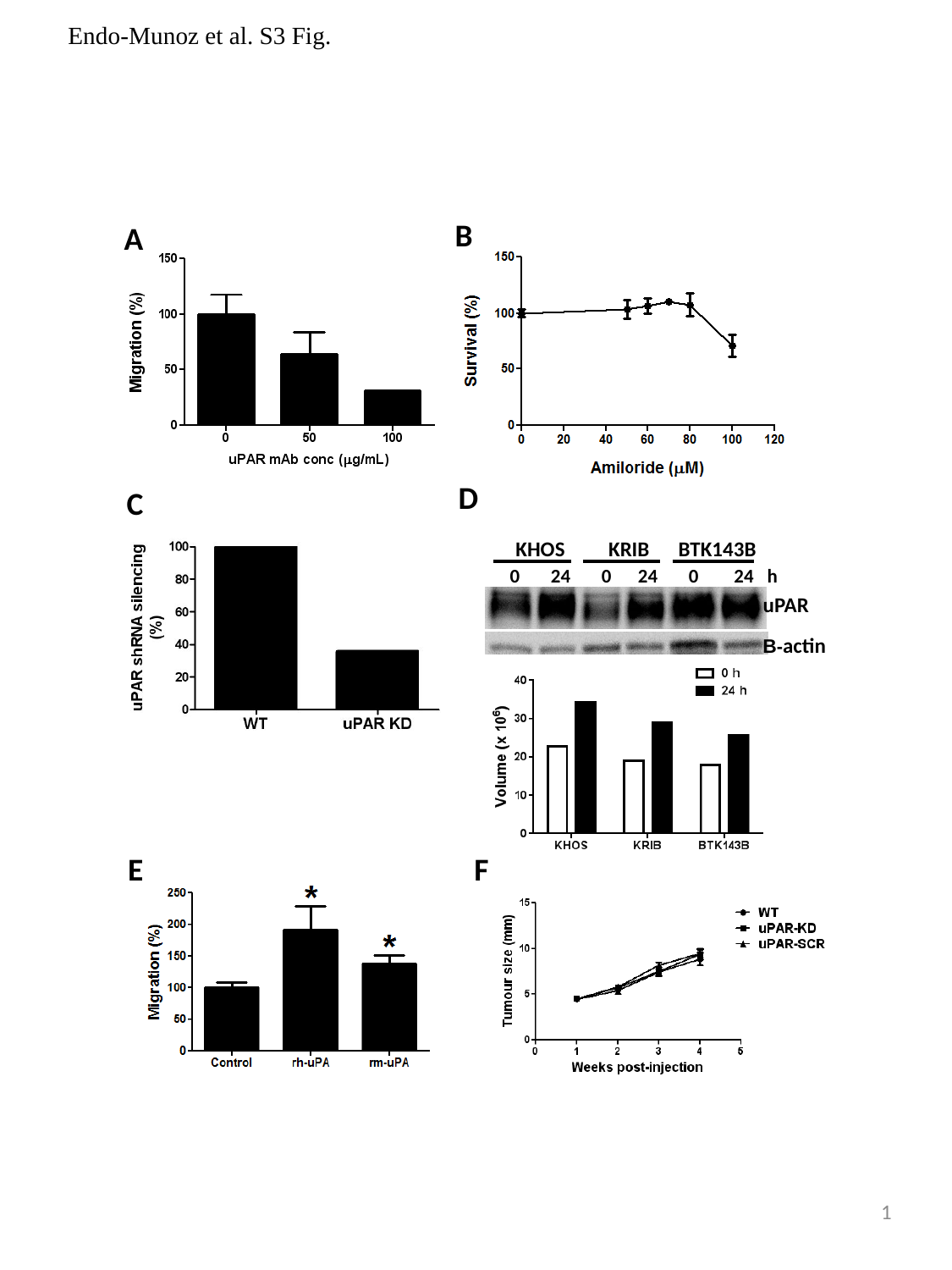

Endo-Munoz et al. S3 Fig.
B
A
D
C
KHOS KRIB BTK143B
0 24 0 24 0 24 h
uPAR
Β-actin
E
F
1

Supplement: S3 Fig — (A) Migration of metastatic KRIB cells in the presence of 0–100 μg/mL of a neutralizing mAb (American Diagnostica) against uPAR. Bars: SEM. Results of at least two experiments in triplicate. (B) Toxicity assay of amiloride in KHOS cells. Assay was performed for 24 h at a cell concentration of 5 x 10^4^ cells/mL using the Cell Titer96 AQueous One Solution Cell Proliferation Assay (Promega). Results of at least two experiments in triplicate. (C) Gene expression (PCR) of uPAR in KHOS cells before (WT) and after shRNA silencing (uPAR-KD). (D) uPAR expression (immunoblotting) in KHOS, KRIB and BTK143B cells after 24 h treatment with 100 nM HMW uPA. Mouse anti-human uPAR (clone 109801) (Santa Cruz), 1:200; Mouse monoclonal anti-human B-actin (C4) (Santa Cruz), 1:5000. Quantitative analysis was performed to correct for B-actin, the detection of which was affected by the WB non-reducing conditions. (E) Migration of KHOS cells in the presence of recombinant human (rh) and recombinant murine (rm) uPA, at 1 μg/mL. Percentage migration is normalized against KHOS control. Results of at least two experiments in triplicate. Bars: SEM. *P < 0.04. (F) Tumour growth, measured as tumour diameter (mm), in mice (n = 5) injected intra-femorally with KHOS WT, uPAR-KD or uPAR-SCR. Bars: SEM. (PPTX) [file pone.0133592.s003.pptx]

## Slide 1
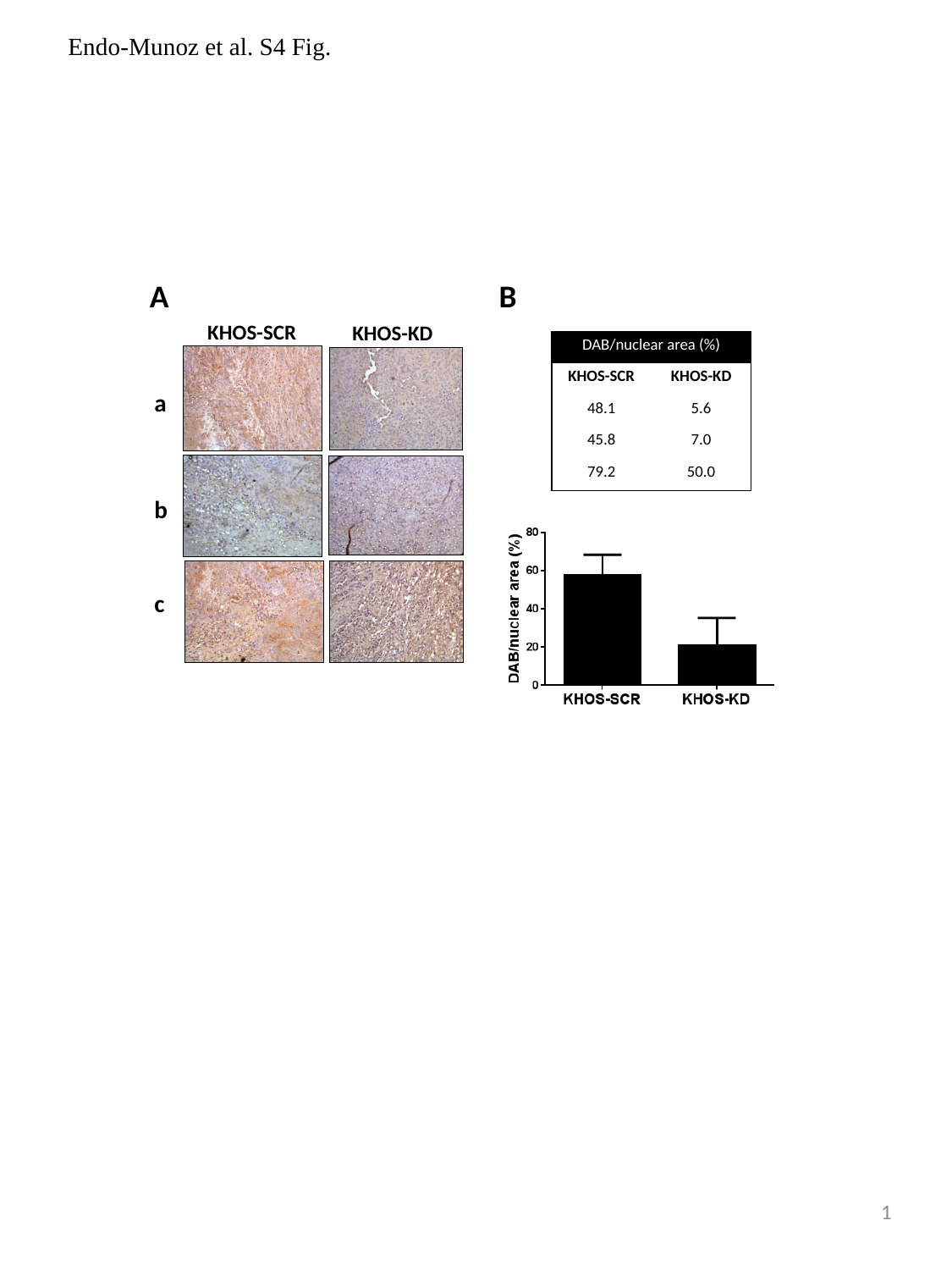

Endo-Munoz et al. S4 Fig.
B
A
KHOS-SCR
KHOS-KD
a
b
c
| DAB/nuclear area (%) | |
| --- | --- |
| KHOS-SCR | KHOS-KD |
| 48.1 | 5.6 |
| 45.8 | 7.0 |
| 79.2 | 50.0 |
1

Supplement: S4 Fig — (A) Representative FFPE tumour sections from different mice (a, b, c) injected with KHOS-SCR (control) or KHOS-KD. IHC using a commercial uPAR antibody (Santa Cruz goat anti-human uPAR, 1:200), and DAB staining (brown). Magnification: 20X. (B) Quantitative image analysis was performed using ImmunoRatio [24]. Bars: SEM. (PPTX) [file pone.0133592.s004.pptx]

## Slide 1
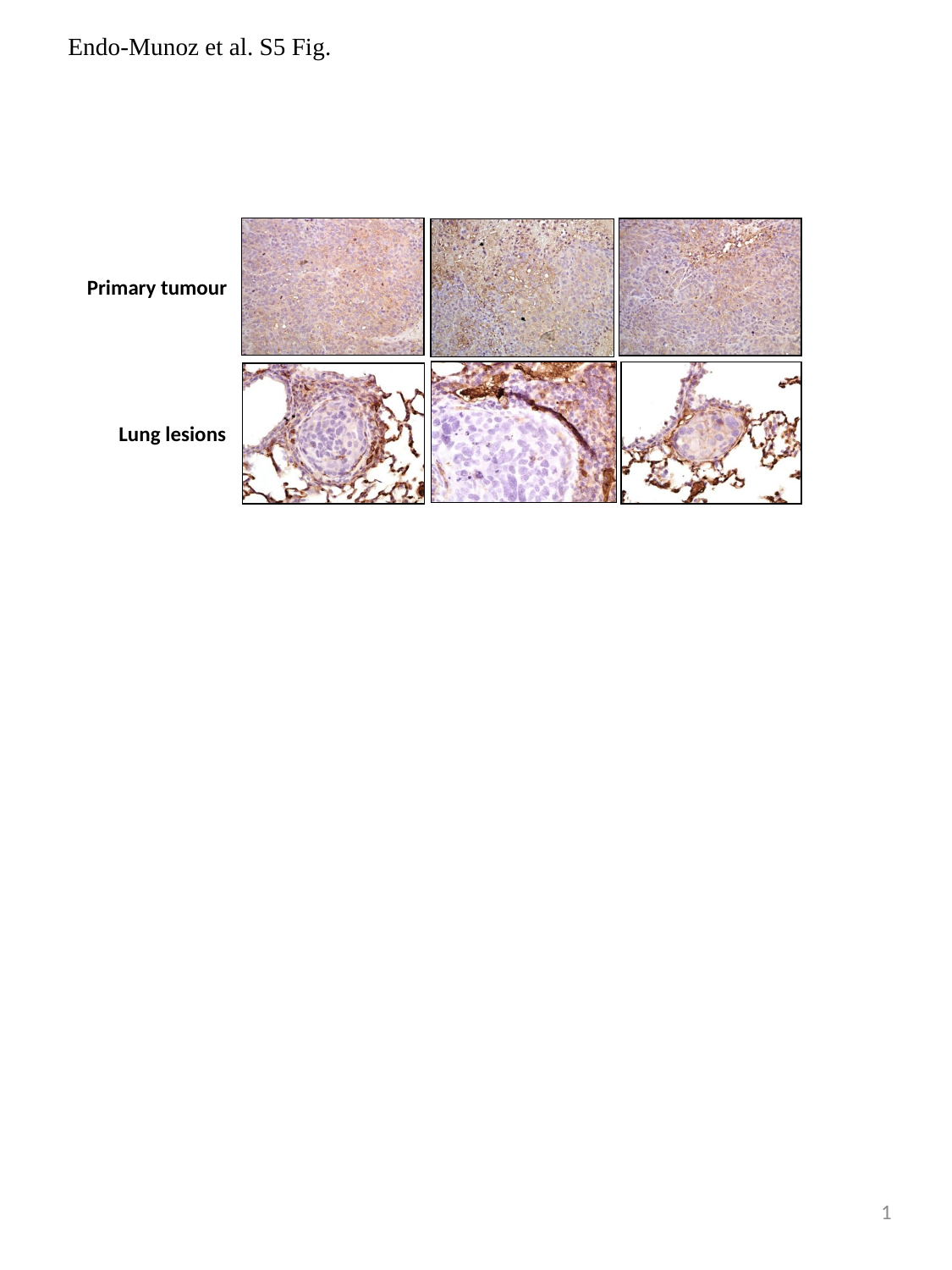

Endo-Munoz et al. S5 Fig.
Primary tumour
Lung lesions
1

Supplement: S5 Fig — Immunohistochemistry in FFPE sections. uPAR antibody (Santa Cruz), 1:200. DAB staining (brown). (PPTX) [file pone.0133592.s005.pptx]
