# Supplementary material for: Progression of Osteosarcoma from a Non-Metastatic to a Metastatic Phenotype Is Causally Associated with Activation of an Autocrine and Paracrine uPA Axis
Source: PLoS One. 2015 Aug 28;10(8):e0133592. doi: 10.1371/journal.pone.0133592 (PMC4552671; doi:10.1371/journal.pone.0133592)
Supplement: S1 Table — Genes upregulated (FC > 2.0, B value > 3.0) in metastatic OS cells compared to non-metastatic OS cells. Transcriptomic analysis performed on Illumina HT-12 Expression BeadChips. (PDF) [file pone.0133592.s006.pdf]

Endo-Munoz et al. Supplementary Table 1

Gene	Name	Accession No.	FC	B value
uPA	urokinase plasminogen activator	NM_002658	8.64	17.14
AHNAK2	AHNAK nucleoprotein 2	NM_024064	8.25	14.22
MLPH	melanophilin	NM_024101	7.08	21.51
IGFBP4	insulin-like growth factor binding protein 4	NM_001552	6.14	15.97
NFKBIA	nuclear factor of kappa light polypeptide gene enhancer in B-cells inhibitor, alpha	NM_020529	6.09	11.48
TMEM158	transmembrane protein 158	NM_015444	6.00	17.33
VANGL2	vang-like 2 (van gogh, Drosophila)	NM_020335	5.86	8.84
TNFRSF21	tumor necrosis factor receptor superfamily, member 21	NM_014452	5.50	13.78
IL11	interleukin 11	NM_000641	5.48	9.81
C13orf15	chromosome 13 open reading frame 15	NM_014059	5.40	18.61
VASN	vasorin	NM_138440	5.24	13.1
CYTL1	cytokine-like 1	NM_018659	5.23	7.26
FGD5	FYVE, RhoGEF and PH domain containing 5	NM_152536	4.96	16.98
RNF150	ring finger protein 150	NM_020724	4.93	12.6
ETFB	electron-transfer-flavoprotein, beta polypeptide	NM_001985	4.75	9.83
ODZ3	odz, odd Oz/ten-m homolog 3 (Drosophila)	NM_001080477	4.70	12.32
MTMR11	myotubularin related protein 11	NM_181873	4.68	15.94
OSTF1	osteoclast stimulating factor 1	NM_012383	4.68	5.54
BCAR3	breast cancer anti-estrogen resistance 3	NM_003567	4.66	20.34
PC-3/MSMP	microseminoprotein, prostate associated	NM_001044264	4.58	13.67
FEZ1	fasciculation and elongation protein zeta 1 (zygin I)	NM_005103	4.56	11.76
RARRES3	retinoic acid receptor responder (tazarotene induced) 3	NM_004585	4.50	4.97
HPCAL1	hippocalcin-like 1	NM_002149	4.49	16.39
LHX6	LIM homeobox 6	NM_199160	4.46	13.77
OSAP	ovary-specific acidic protein	NM_032623	4.45	11.93
LZTS1	leucine zipper, putative tumor suppressor 1	NM_021020	4.32	7.94
SYK	spleen tyrosine kinase	NM_003177	4.13	5.28
ZSCAN18	zinc finger and SCAN domain containing 18	NM_023926	4.09	13.93
DLX5	distal-less homeobox 5	NM_005221	4.08	14.44
EVL	Enah/Vasp-like	NM_016337	3.95	6.29
ETV5	est variant 5	NM_004454	3.94	8.93
CRMP1	collapsin response mediator protein 1	NM_001313	3.84	13.57
TTC15	tetratricopeptide repeat domain 15	NM_016030	3.80	9.79
GABARAPL1	GABA(A) receptor-associated protein like 1	NM_031412	3.80	6.32
CBLB	Cas-Br-M (murine) ecotropic retroviral transforming sequence b	NM_170662	3.68	6.21
KANK1	KN motif and ankyrin repeat domains 1	NM_153186	3.67	7.46
GYPC	glycophorin C (Gerbich blood group)	NM_002101	3.54	7.83
LRRC33	leucine rich repeat containing 33	NM_198565	3.51	10.19
NRP1/VEGF165R	neuropilin 1	NM_003873	3.51	6.64
PRMT2	protein arginine methyltransferase 2	NM_001535	3.50	5.25
FAM43A	family with sequence similarity 43, member A	NM_153690	3.50	7.46
ZIC2	Zic family member 2 (odd-paired homolog, Drosophila)	NM_007129	3.39	7.15
RPL26L1	Ribosomal protein L26-like 1	NM_016093	3.39	16.26
UNC45A	unc-45 homolog A (C. elegans)	NM_018671	3.33	3.7
IRF8	interferon regulatory factor 8	NM_002163	3.32	15.74
C14orf159	chromosome 14 open reading frame 159	NM_024952	3.31	8.12
CYGB	cytoglobin	NM_134268	3.27	5.74
TGFBR2	transforming growth factor, beta receptor II (70/80kDa)	NM_003242	3.24	6.03
ELP2	elongation protein 2 homolog (S. cerevisiae)	NM_018255	3.23	8.62
COL1A2	collagen, type I, alpha 2	NM_000089	3.23	13.64
CD68	CD68 molecule	NM_001251	3.19	5.63
NDN	necdin homolog (mouse)	NM_002487	3.17	12.76
MAMLD1	mastermind-like domain containing 1	NM_005491	3.16	7.98
MFGE8	milk fat globule-EGF factor 8 protein	NM_005928	3.10	11.13
CCDC81	coiled-coil domain containing 81	NM_021827	3.09	4.80
ISG20	interferon stimulated exonuclease gene 20kDa	NM_002201	3.05	6.49
MAP1A	microtubule-associated protein 1A	NM_002373	3.05	5.99
ATG16L1	ATG16 autophagy related 16-like 1 (S. cerevisiae)	NM_198890	3.00	3.79
LARGE	like-glycosyltransferase	NM_004737	2.99	12.12
CCDC167	coiled-coil domain containing 167	NM_138493	2.98	9.56
NKD2	naked cuticle homolog 2	NM_033120	2.94	5.85
RCC2	regulator of chromosome condensation 2	NM_018715	2.93	5.81
BEX5	brain expressed, X-linked 5	NM_001012978	2.92	5.73
CEBPD	CCAAT/enhancer binding protein (C/EBP), delta	NM_005195	2.91	8.95
FAM65B	family with sequence similarity 65, member B	NM_015864	2.89	12.98
TMEFF2	transmembrane protein with EGF-like and two follistatin-like domains 2	NM_016192	2.89	10.77
C14orf132	chromosome 14 open reading frame 132	NM_023938	2.88	8.09

CLDN1	claudin 1	NM_021101	2.87	6.01
SLC2A3	solute carrier family 2 (facilitated glucose transporter), member 3	NM_006931	2.83	6.91
SELS	selenoprotein S	NM_203472	2.81	5.9
CLPTM1L	CLPTM1-like	NM_030782	2.80	8.53
TFDP1	transcription factor DP-1	NM_007111	2.77	4.13
LETMD1	LETM1 domain containing 1	NM_015416	2.76	3.71
SP8	SP8 transcription factor	NM_182700	2.73	4.27
TSGA14	testis specific, 14	NM_018718	2.73	5.74
STOX2	storkhead box 2	NM_020225	2.73	9.03
FAM89A	family with sequence similarity 89, member A	NM_198552	2.72	6.34
KCNJ2	potassium inwardly-rectifying channel, subfamily J, member 2	NM_000891	2.71	10.24
TLN2	talin 2	NM_015059	2.71	6.49
ITPR3	inositol 1,4,5-triphosphate receptor, type 3	NM_002224	2.69	11.13
RPL26L1	ribosomal protein L26-like 1	NM_016093	2.69	17.39
HOXC6	homeobox C6	NM_004503	2.68	6.82
PROK2	prokineticin 2	NM_021935	2.67	14.12
FOXD2	forkhead box D2	NM_004474	2.67	3.89
HSPB3	heat shock 27kDa protein 3	NM_006308	2.63	11.55
C6orf52	chromosome 6 open reading frame 52	NM_001145020	2.62	6.16
BMP4	bone morphogenetic protein 4	NM_001202	2.61	9.18
IGSF3	immunoglobulin superfamily, member 3	NM_001542	2.60	6.45
ZNF792	zinc finger protein 792	NM_175872	2.57	9.82
HOPX	HOP homeobox	NM_139211	2.55	16.12
MSLN	mesothelin	NM_005823	2.55	3.46
NGRN	neugrin, neurite outgrowth associated	NM_001033088	2.55	3.66
NOS2A	nitric oxide synthase 2, inducible	NM_000625	2.54	3.84
SMAD6	SMAD family member 6	NM_005585	2.54	6.83
TCEAL7	transcription elongation factor A (SII)-like 7	NM_152278	2.52	4.05
SLC25A4	solute carrier family 25 (mitochondrial carrier; adenine nucleotide translocator), member 4	NM_001151	2.51	3.93
CDH4	cadherin 4, type 1, R-cadherin	NM_001794	2.51	9.28
ADORA2A	adenosine A2a receptor	NM_000675	2.47	10.09
MXRA7	matrix-remodelling associated 7	NM_198530	2.47	5.76
EFHD2	EF-hand domain family, member D2	NM_024329	2.46	6.92
LEPREL1	leprecan-like 1	NM_018192	2.45	8.51
ATP5A1	ATP synthase, H+ transporting, mitochondrial F1 complex, alpha subunit 1	NM_004046	2.41	10.03
POLG	polymerase (DNA directed), gamma	NM_002693	2.39	4.37
KHDRBS3	KH domain containing, RNA binding, signal transduction associated 3	NM_006558	2.37	5.38
STK40	serine/threonine kinase 40	NM_032017	2.36	7.88
SNAI2/SLUG	snail homolog 2	NM_003068	2.33	7.12
SLC27A2	solute carrier family 27 (fatty acid transporter), member 2	NM_003645	2.31	8.89
CTDSPL	CTD (carboxy-terminal domain, RNA polymerase II, polypeptide A) small phosphatase-like	NM_005808	2.27	4.53
DSE	dermatan sulfate epimerase	NM_013352	2.27	8.16
LGMN	legumain	NM_005606	2.26	7.83
CCNA1	cyclin A1	NM_001237	2.25	4.36
SH3GL2	SH3-domain GRB2-like 2	NM_003026	2.24	14.7
TLE4	transducin-like enhancer of split 4 (E(sp1) homolog, Drosophila)	NM_007005	2.24	3.19
ARID3B	AT rich interactive domain 3B (BRIGHT-like)	NM_006465	2.22	9.16
NUAK1	NUAK family, SNF1-like kinase, 1	NM_014840	2.22	10.17
SH3RF2	SH3 domain containing ring finger 2	NM_152550	2.21	3.64
IRX2	iroquois homeobox 2	NM_033267	2.19	15.05
TEK	TEK tyrosine kinase, endothelial	NM_000459	2.17	8.41
LMBR1	limb region 1 homolog (mouse)	NM_022458	2.17	7.05
KRT80	keratin 80	NM_182507	2.16	9.75
SLC20A1	solute carrier family 20 (phosphate transporter), member 1	NM_005415	2.15	6.40
MUTYH	mutY homolog (E. coli)	NM_012222	2.15	8.98
PTPRM	protein tyrosine phosphatase, receptor type, M	NM_002845	2.14	6.16
DHCR7	7-dehydrocholesterol reductase	NM_001360	2.13	8.04
JAM3	junctional adhesion molecule 3	NM_032801	2.12	4.89
TNFRSF9	tumor necrosis factor receptor superfamily, member 9	NM_001561	2.09	3.12
AGK	acylglycerol kinase	NM_018238	2.07	11.04
HSD17B2	Hydroxysteroid (17-beta) dehydrogenase 2	NM_002153	2.05	6.48
F2RL3	coagulation factor II (thrombin) receptor-like 3	NM_003950	2.05	4.62
SLC44A2	solute carrier family 44, member 2	NM_020428	2.03	3.79
RBP1	retinol binding protein 1, cellular	NM_002899	2.02	10.47
ARHGAP17	Rho GTPase activating protein 17	NM_018054	2.02	3.02
OTUB2	OTU domain, ubiquitin aldehyde binding 2	NM_023112	2.01	8.77
STIP1	stress-induced-phosphoprotein 1	NM_006819	2.00	3.16
