# Supplementary material for: Progression of Osteosarcoma from a Non-Metastatic to a Metastatic Phenotype Is Causally Associated with Activation of an Autocrine and Paracrine uPA Axis
Source: PLoS One. 2015 Aug 28;10(8):e0133592. doi: 10.1371/journal.pone.0133592 (PMC4552671; doi:10.1371/journal.pone.0133592)
Supplement: S2 Table — Proteins present on the plasma membrane of metastatic OS cells compared to non-metastatic OS cells. Quantitative proteomic analysis was performed using SILAC labelling. Heavy (metastatic OS cells) and light (non-metastatic OS cells) lysates were mixed 1:1 by protein weight and then separated by 10% SDS-PAGE before LC-MS/MS analysis. (PDF) [file pone.0133592.s007.pdf]

Endo-Munoz et al. Supporting Information Table 2

Accession No.	Symbol	Entry Name	Heavy:Light Ratio
**Q03405**	**PLAUR**	**Urokinase plasminogen activator surface receptor**	**100.36**
P61970	NUTF2	Nuclear transport factor 2	31.17
P61803	DAD1	Defender against cell death	28.76
Q96BD0	SLCO4A1	Solute carrier organic anion transporter family member 4A1	24.13
Q9UM22	EPDR1	Mammalian ependymin-related protein 1	23.48
Q6IAN0	DHRS7B	Dehydrogenase/reductase SDR family member 7B	14.10
Q9H5V8	CDCP1	CUB domain-containing protein 1	10.16
O14786	NRP1	Neuropilin-1	9.35
P35237	SERPINB6	Serpin B6	7.13
Q9C004	SPRY4	Protein sprouty homolog 4	6.52
P07858	CTSB	Cathepsin B	5.90
O95182	NDUFA7	NADH dehydrogenase [ubiquinone] 1 alpha subcomplex subunit 7	5.63
P17900	GM2A	Ganglioside GM2 activator	3.98
Q16698	DECR1	2,4-dienoyl-CoA reductase, mitochondrial	3.69
O15258	RER1	Protein RER1	3.21
Q9P2E9	RRBP1	Ribosome-binding protein 1	3.14
Q16678	CYP1B1	Cytochrome P450 1B1	2.99
P34059	GALNS	N-acetylgalactosamine-6-sulfatase	2.95
O00592	PODXL	Podocalyxin-like protein 1	2.94
P07602	PSAP	Proactivator polypeptide 2	2.86
Q96S66	CLCC1	Chloride channel CLIC-like protein 1	2.80
P16278	GLB1	Beta-galactosidase	2.77
P17096	HMGA1	High mobility group protein HMG-I/HMG-Y	2.73
P22307	SCP2	Non-specific lipid-transfer protein	2.73
Q6PI48	DARS2	Aspartyl-tRNA synthetase, mitochondrial	2.70
P11310	ACADM	Medium-chain specific acyl-CoA dehydrogenase, mitochondrial	2.66
Q96IR7	HPDL	4-hydroxyphenylpyruvate dioxygenase-like protein	2.56
Q15165	PON2	Serum paraoxonase/arylesterase 2	2.54
O00299	CLIC1	Chloride intracellular channel protein 1	2.53
Q6PIU2	NCEH1	Neutral cholesterol ester hydrolase 1	2.48
O75947	ATP5H	ATP synthase subunit d, mitochondrial	2.42
P61019	RAB2A	Ras-related protein Rab-2A	2.42
P61421	ATP6V0D1	V-type proton ATPase subunit d 1	2.42
Q4KWH8	PLCH1	1-phosphatidylinositol-4,5-bisphosphate phosphodiesterase eta-1	2.36
P51572	BCAP31	B-cell receptor-associated protein 31	2.32
Q04837	SSBP1	Single-stranded DNA-binding protein, mitochondrial	2.26
Q8NBJ7	SUMF2	Sulfatase-modifying factor 2	2.26
P50281	MMP14	Matrix metalloproteinase-14	2.24
Q13641	TPBG	Trophoblast glycoprotein =1	2.19
P13667	PDIA4	Protein disulfide-isomerase A4	2.16
O95831	AIFM1	Apoptosis-inducing factor 1, mitochondrial	2.15
Q9H3G5	CPVL	Probable serine carboxypeptidase CPVL	2.15
Q8IXB1	DNAJC10	DnaJ homolog subfamily C member 10	2.11
P24539	ATP5F1	ATP synthase subunit b, mitochondrial	2.07
Q9Y6N5	SQRDL	Sulfide:quinone oxidoreductase, mitochondrial	2.07
P56134	ATP5J2	ATP synthase subunit f, mitochondrial	2.01
