# Supplementary material for: Progression of Osteosarcoma from a Non-Metastatic to a Metastatic Phenotype Is Causally Associated with Activation of an Autocrine and Paracrine uPA Axis
Source: PLoS One. 2015 Aug 28;10(8):e0133592. doi: 10.1371/journal.pone.0133592 (PMC4552671; doi:10.1371/journal.pone.0133592)
Supplement: S3 Table — Upregulated genes (FC > 2.0, B > 3.0) in metastatic OS cells compared to non-metastatic OS cells, after 24 h treatment with bone marrow cell conditioned medium. Transcriptomic analysis performed on Illumina HT-12 Expression BeadChips. (PDF) [file pone.0133592.s008.pdf]

Endo-Munoz et al. Supporting Information Table 3

Gene	Description	Accession No.	FC	B value
TMEM158	transmembrane protein 158	NM_015444	8.49	9.52
**PLAU**	**urokinase plasminogen activator**	**NM_002658**	**7.97**	**18.82**
PHLDA1	pleckstrin homology-like domain, family A, member 1	NM_007350	7.58	14.81
MTMR11	myotubularin related protein 11	NM_181873	7.02	15.08
DUSP5	dual specificity phosphatase 5	NM_004419	6.57	12.78
CYBA	cytochrome b-245, alpha polypeptide	NM_000101	6.50	10.54
MLPH	melanophilin	NM_024101	6.42	20.20
PRDM8	PR domain containing 8	NM_020226	6.40	21.35
CD68	CD68 molecule	NM_001251	6.01	15.48
CTHRC1	collagen triple helix repeat containing 1	NM_138455	5.98	8.22
STX1A	syntaxin 1A (brain)	NM_004603	5.94	15.54
IGF2BP3	insulin-like growth factor 2 mRNA binding protein 3	NM_006547	5.73	8.82
EMP1	epithelial membrane protein 1	NM_001423	5.48	8.99
HPCAL1	hippocalcin-like 1	NM_002149	5.47	15.71
OSCAR	osteoclast associated, immunoglobulin-like receptor	NM_133169	5.46	6.19
TNFRSF19	tumor necrosis factor receptor superfamily, member 19	NM_018647	5.30	9.66
PLTP	phospholipid transfer protein	NM_006227	5.30	11.02
TFPI	tissue factor pathway inhibitor (lipoprotein-associated coagulation inhibitor)	NM_006287	5.30	12.66
S100A16	S100 calcium binding protein A16	NM_80388	5.27	9.65
RGS17	regulator of G-protein signaling 17	NM_012419	5.23	14.96
TGFBR2	transforming growth factor, beta receptor II (70/80kDa)	NM_003242	5.21	9.95
RAGE	renal tumour antigen	NM_014226	5.20	7.32
CARD8	caspase recruitment domain family, member 8	NM_014959	5.15	18.28
ANTXR2	anthrax toxin receptor 2	NM_058172	5.02	17.13
ERRFI1	ERBB receptor feedback inhibitor 1	NM_018948	5.01	7.51
BCAR3	breast cancer anti-estrogen resistance 3	NM_003567	4.91	9.21
HMGA1	high mobility group AT-hook 1	NM_002131	4.78	12.57
SPRY4	sprouty homolog 4 (Drosophila)	NM_030964	4.77	10.04
STC1	stanniocalcin 1	NM_003155	4.74	3.11
TNFRSF21	tumor necrosis factor receptor superfamily, member 21	NM_014452	4.74	6.86
COL6A3	collagen, type VI, alpha 3	NM_004369	4.70	5.26
IGFBP4	insulin-like growth factor binding protein 4	NM_001552	4.63	4.51
FAM129A	family with sequence similarity 129, member A	NM_052966	4.62	5.79
SLC20A1	solute carrier family 20 (phosphate transporter), member 1	NM_005415	4.62	10.61
C13orf15	chromosome 13 open reading frame 15	NM_014059	4.58	11.49
MSX1	Msh homeobox1	NM_002448	4.57	7.10
HOPX	HOP homeobox	NM_139211	4.53	12.05
FKBP11	FK506 binding protein 11, 19 kDa	NM_016594	4.51	6.29
FOSL1	FOS-like antigen 1	NM_005438	4.50	3.20
GFPT2	glutamine-fructose-6-phosphate transaminase 2	NM_005110	4.43	10.40
PLCH2	phospholipase C, eta 2	NM_014638	4.43	8.68
NPC1	Niemann-Pick disease, type C1	NM_000271	4.42	14.43
SH2B3	SH2B adaptor protein 3	NM_005475	4.40	9.38
FOXA2	forkhead box A2	NM_021784	4.38	12.88
SLCO4A1	solute carrier organic anion transporter family, member 4A1	NM_016354	4.36	4.77
HEY1	hairy/enhancer-of-split related with YRPW motif 1	NM_012258	4.35	15.68
PLEK2	plekstrin 2	NM_016445	4.31	12.61
NRP1	neuropilin 1	NM_003873	4.25	14.16
COL7A1	collagen, type VII, alpha 1	NM_000094	4.17	6.15
NTSR1	neurotensin receptor 1 (high affinity)	NM_002531	4.16	17.46
GMPPB	neurotensin receptor 1 (high affinity)	NM_013334	4.14	4.33
SPRED1	sprouty-related, EVH1 domain containing 1	NM_152594	3.90	6.30
DKK1	dickkopf homolog 1 (Xenopus laevis)	NM_012242	3.88	6.40
FGD5	FYVE, RhoGEF and PH domain containing 5	NM_152536	3.84	14.17
RAP1GAP2	RAP1 GTPase activating protein 2	NM_015085	3.84	12.44
ITPR3	inositol 1,4,5-trisphosphate receptor, type 3	NM_002224	3.84	12.25
TRIB1	tribbles homolog 1 (Drosophila)	NM_025195	3.83	12.43
RAC2	Ras-related C3 botulinum toxin substrate 2 (rho family, small GTP binding protein Rac2)	NM_002872	3.74	15.48
MAP1LC3A	microtubule-associated protein 1 light chain 3 alpha	NM_181509	3.73	13.05
RNF149	ring finger protein 149	NM_173647	3.73	9.01
BAIAP2L2	BAI1-associated protein 2-like 2	NM_025045	3.66	5.40
KLF2	Kruppel-like factor 2 (lung)	NM_016270	3.64	3.72
ZNF581	zinc finger protein 581	NM_016535	3.61	9.46
FOXR1	forkhead box R1	NM_181721	3.60	3.70
AKAP12	A kinase (PRKA) anchor protein 12	NM_005100	3.60	11.92
FAM20C	family with sequence similarity 20, member C	NM_020223	3.59	4.18
BNIP3	BCL2/adenovirus E1B 19kDa interacting protein 3	NM_004052	3.59	6.12
PLBD1	phospholipase B domain containing 1	NM_024829	3.57	9.53
WLS	Wntless homolog (Drosophila)	NM_024911	3.56	5.66
FEZ2	fasciculation and elongation protein zeta 2 (zygin II)	NM_005102	3.56	3.73
CLDN7	claudin 7	NM_001307	3.53	13.11

RPL17	ribosomal protein L17	NM_000985	3.50	9.96
RPS7	ribosomal protein S7	NM_001011	3.50	5.09
SPIRE1	spire homolog 1 (Drosophila)	NM_020148	3.49	7.67
DDX10	DEAD (Asp-Glu-Ala-Asp) box polypeptide 10	NM_004398	3.48	11.38
TNFRSF25	tumor necrosis factor receptor superfamily, member 25	NM_003790	3.47	9.65
SLC4A11	solute carrier family 4, sodium borate transporter, member 11	NM_032034	3.45	4.32
**PLAUR**	**plasminogen activator, urokinase receptor**	**NM_002659**	**3.43**	**6.93**
IL11	interleukin 11	NM_000641	3.41	10.03
BCL2L13	BCL2-like 13 (apoptosis facilitator)	NM_015367	3.33	6.90
TOX2	TOX high mobility group box family member 2	NM_032883	3.33	5.08
WLS	Wntless homolog (Drosophila)	NM_024911	3.31	4.31
SLC4A7	solute carrier family 4, sodium bicarbonate cotransporter, member 7	NM_003615	3.30	6.13
KDSR	3-ketodihydrosphingosine reductase	NM_002035	3.27	13.48
DYNC1H1	dynein, cytoplasmic 1, heavy chain 1	NM_001376	3.26	5.31
ITGA2	integrin, alpha 2 (CD49B, alpha 2 subunit of VLA-2 receptor)	NM_002203	3.24	14.99
RGS2	regulator of G-protein signaling 2, 24kDa	NM_002923	3.23	8.63
C12orf24	chromosome 12 open reading frame 24	NM_013300	3.22	10.04
TSC22D1	TSC22 domain family, member 1	NM_006022	3.22	9.22
ADCY1	adenylate cyclase 1 (brain)	NM_021116	3.20	10.01
KLF4	Kruppel-like factor 4 (gut)	NM_004235	3.18	11.00
MT1G	metallothionein 1G	NM_005950	3.16	9.09
FBXO32	F-box protein 32	NM_058229	3.16	5.99
TGIF1	TGFB-induced factor homeobox 1	NM_003244	3.13	10.52
SPRY2	sprouty homolog 2 (Drosophila)	NM_005842	3.12	9.42
RTCD1	RNA terminal phosphate cyclase domain 1	NM_003729	3.12	5.55
PITPNM1	phosphatidylinositol transfer protein, membrane-associated 1	NM_004910	3.12	5.97
KLF6	Kruppel-like factor 6	NM_001300	3.08	4.21
KIAA1949	KIAA1949	NM_133479	3.07	6.80
ARHGAP4	Rho GTPase activating protein 4	NM_001666	3.06	12.54
RBPJ	recombination signal binding protein for immunoglobulin kappa J region	NM_005349	3.06	8.72
FLJ20444	hypothetical protien FLJ20444	XM_003119170	3.05	3.23
NET1	TGFB-induced factor homeobox 1	NM_005863	3.01	8.87
WBSCR27	Williams Beuren syndrome chromosome region 27	NM_152559	3.00	12.67
STAMBPL1	STAM binding protein-like 1	NM_020799	2.99	3.95
PVRL3	poliovirus receptor-related 3	NM_015480	2.99	11.44
CDRT4	CMT1A duplicated region transcript 4	NM_173622	2.98	7.90
SH3KBP1	SH3-domain kinase binding protein 1	NM_031892	2.94	15.44
MAP4K4	mitogen-activated protein kinase kinase kinase kinase 4	NM_004834	2.94	8.43
OBFC1	oligonucleotide/oligosaccharide-binding fold containing 1	NM_024928	2.92	11.73
KCNMA1	potassium large conductance calcium-activated channel, subfamily M, alpha member 1	NM_002247	2.91	3.38
MUTYH	MutY homolog (E. coli)	NM_012222	2.91	6.87
DPP9	dipeptidyl-peptidase 9	NM_139159	2.88	10.20
TM4SF19	transmembrane 4 L six family member 19	NM_138461	2.88	7.14
ACADVL	acyl-CoA dehydrogenase, very long chain	NM_000018	2.87	16.47
SULF2	sulfatase 2	NM_018837	2.85	5.22
CAMLG	calcium modulating ligand	NM_001745	2.85	3.99
CRIP2	cysteine-rich protein 2	NM_001312	2.76	13.07
LPXN	leupaxin	NM_004811	2.75	9.02
ADAM8	ADAM metallopeptidase domain 8	NM_001109	2.75	3.41
STXBP3	ADAM metallopeptidase domain 8	NM_007269	2.73	9.49
RGS20	regulator of G-protein signaling 20	NM_003702	2.73	8.93
UBE2E3	ubiquitin-conjugating enzyme E2E 3	NM_182678	2.73	8.42
CTSB	cathepsin B	NM_001908	2.71	4.57
TWF2	twinfilin, actin-binding protein, homolog 2 (Drosophila)	NM_007284	2.71	8.70
NUMB	Numb homolog (Drosophila)	NM_003744	2.70	3.82
TAGLN3	transgelin 3	NM_013259	2.69	12.56
APRT	adenine phosphoribosyltransferase	NM_000485	2.68	6.16
AHNAK2	AHNAK nucleoprotein 2	NM_138420	2.66	5.79
PTDSS1	phosphatidylserine synthase 1	NM_014754	2.65	8.06
TMBIM4	transmembrane BAX inhibitor motif containing 4	NM_016056	2.65	3.65
NMD3	NMD3 homolog (S. cerevisiae)	NM_015938	2.63	6.89
CACNG6	calcium channel, voltage-dependent, gamma subunit 6	NM_031897	2.62	4.24
LETMD1	LETM1 domain containing 1	NM_015416	2.62	5.64
VIM	vimentin	NM_003380	2.59	8.31
GSTM1	glutathione S-transferase mu 1	NM_000561	2.59	5.12
TACC2	transforming, acidic coiled-coil containing protein 2	NM_006997	2.58	5.35
EDG7	lysophosphatidic acid receptor 3	NM_012152	2.58	8.70
SSU72	SSU72 RNA polymerase II CTD phosphatase homolog (S. cerevisiae)	NM_014188	2.57	4.19
TBC1D8	TBC1 domain family, member 8 (with GRAM domain)	NM_001102426	2.55	7.25
FAM129B	family with sequence similarity 129, member B	NM_022833	2.52	9.14
GLRX	glutaredoxin (thioltransferase)	NM_002064	2.51	3.60
DUSP4	dual specificity phosphatase 4	NM_057158	2.51	3.27
CHIC2	cysteine-rich hydrophobic domain 2	NM_012110	2.50	3.47
FRMD4A	FERM domain containing 4A	NM_018027	2.49	8.65

TMEM42	FERM domain containing 4A	NM_144638	2.49	3.99
ACP6	acid phosphatase 6, lysophosphatidic	NM_016361	2.48	9.24
LPIN2	lipin 2	NM_014646	2.47	6.09
FXYD5	FXYD domain containing ion transport regulator 5	NM_014164	2.46	13.16
SOX9	SRY (sex determining region Y)-box 9	NM_000346	2.46	7.22
PITPNC1	phosphatidylinositol transfer protein, cytoplasmic 1	NM_181671	2.46	11.16
MSN	moesin	NM_002444	2.45	7.42
SLC25A24	solute carrier family 25 (mitochondrial carrier; phosphate carrier), member 24	NM_013386	2.42	8.84
TUBGCP2	solute carrier family 25 (mitochondrial carrier; phosphate carrier), member 24	NM_006659	2.39	6.11
PRSS3	protease, serine, 3	NM_002771	2.39	13.77
RER1	RER1 retention in endoplasmic reticulum 1 homolog (S. cerevisiae)	NM_007033	2.37	10.98
ARHGEF19	Rho guanine nucleotide exchange factor (GEF) 19	NM_153213	2.37	5.23
TSSC1	tumor suppressing subtransferable candidate 1	NM_003310	2.36	9.80
RGS10	regulator of G-protein signaling 10	NM_002925	2.35	3.89
C4orf14	chromosome 4 open reading frame 14	NM_032313	2.35	5.56
MT1E	chromosome 4 open reading frame 14	NM_175617	2.34	5.86
HACL1	2-hydroxyacyl-CoA lyase 1	NM_012260	2.34	4.62
TUBB3	tubulin, beta 3	NM_006086	2.33	7.70
MANSC1	MANSC domain containing 1	NM_018050	2.32	13.88
MKRN1	makorin ring finger protein 1	NM_013446	2.31	11.12
FRMD3	FERM domain containing 3	NM_174938	2.30	13.34
CDCP1	CUB domain containing protein 1	NM_178181	2.30	7.03
F2RL1	coagulation factor II (thrombin) receptor-like 1	NM_005242	2.30	8.30
TTC15	tetratricopeptide repeat domain 15	NM_016030	2.30	13.22
GOLPH3L	Golgi phosphoprotein 3-like	NM_018178	2.30	3.94
UTP14A	UTP14, U3 small nucleolar ribonucleoprotein, homolog A (yeast)	NM_006649	2.29	3.82
TMEM156	transmembrane protein 156	NM_024943	2.28	7.71
SEMA3A	Sema domain, immunoglobulin domain (Ig), short basic domain, secreted, (semaphorin) 3A	NM_006080	2.28	8.72
SCG5	secretogranin V (7B2 protein)	NM_003020	2.28	6.71
IDS	iduronate 2-sulfatase	NM_000202	2.28	12.64
FNTA	farnesyltransferase, CAAX box, alpha	NM_002027	2.26	3.94
UBTD1	ubiquitin domain containing 1	NM_024954	2.25	5.44
SVIL	supervillin	NM_003174	2.25	7.99
SNX5	sorting nexin 5	NM_014426	2.24	3.91
DPH5	DPH5 homolog (S. cerevisiae)	NM_015958	2.24	8.78
C12orf56	chromosome 12 open reading frame 56	NM_001099676	2.24	9.23
TMEM204	transmembrane protein 204	NM_024600	2.23	12.93
FAM176B	family with sequence similarity 176, member B	NM_018166	2.21	7.24
C9orf103	chromosome 9 open reading frame 103	NM_001001551	2.21	6.43
SS18	synovial sarcoma translocation, chromosome 18	NM_005637	2.19	3.48
MT1P3	metallothionein 1 pseudogene 3	NM_080757	2.18	3.84
ULK1	Unc-51-like kinase 1 (C. elegans)	NM_003565	2.17	11.46
AGPAT9	1-acylglycerol-3-phosphate O-acyltransferase 9	NM_032717	2.17	6.21
MILR1	mast cell immunoglobulin-like receptor 1	NM_001085423	2.16	5.49
CTBS	chitobiase, di-N-acetyl-	NM_004388	2.12	3.35
DHPS	deoxyhypusine synthase	NM_001930	2.12	11.42
TMEM69	transmembrane protein 69	NM_016486	2.11	8.96
ETV4	Ets variant 4	NM_001986	2.11	5.70
FER1L3	myoferlin	NM_013451	2.10	3.88
TOMM70A	translocase of outer mitochondrial membrane 70 homolog A (S. cerevisiae)	NM_014820	2.10	5.06
TBXAS1	thromboxane A synthase 1 (platelet)	NM_001061	2.09	14.47
LRRC33	leucine rich repeat containing 33	NM_198565	2.08	8.10
EGFR	epidermal growth factor receptor	NM_005228	2.08	7.01
ABR	active BCR-related gene	NM_001092	2.05	3.88
DCBLD2	discoidin, CUB and LCCL domain containing 2	NM_080927	2.04	10.01
LAPTM5	lysosomal protein transmembrane 5	NM_006762	2.04	5.62
RAB31	RAB31, member RAS oncogene family	NM_006868	2.04	7.33
TPST1	tyrosylprotein sulfotransferase 1	NM_003596	2.04	11.99
TM4SF18	transmembrane 4 L six family member 18	NM_138786	2.02	14.87
ATG16L1	ATG16 autophagy related 16-like 1 (S. cerevisiae)	NM_198890	2.01	3.84
EFCAB4A	EF-hand calcium binding domain 4A	NM_173584	2.00	5.79
NMRAL1	NmrA-like family domain containing 1	NM_020677	2.00	3.86
ZDHHC9	zinc finger, DHHC-type containing 9	NM_016032	2.00	3.16
